# Exploring Reversible Redox Behavior in the 6H-BaFeO_3−δ_ (0 < δ < 0.4) System: Impact of Fe^3+^/Fe^4+^ Ratio on CO Oxidation

**DOI:** 10.1021/acs.inorgchem.4c00917

**Published:** 2024-04-29

**Authors:** D. Gutiérrez-Martín, A. Varela, M. Hernando, A. Torres-Pardo, E. Matesanz, I. Gómez-Recio, J. M. González-Calbet, M. T. Fernández-Díaz, J. J. Calvino, M. A. Cauqui, M. P. Yeste, M. Parras

**Affiliations:** † Departamento de Química Inorgánica, Facultad de Ciencias Químicas, Universidad Complutense, 28040 Madrid, Spain; ‡ Unidad de Difracción de Rayos X. Centro de Asistencia a la Investigación de Técnicas Químicas, Universidad Complutense de Madrid, 28040 Madrid, Spain; § Institut Laue-Langevin, 38042 Grenoble cedex 9, France; ∥ Departamento de Ciencia de los Materiales e Ingeniería Metalúrgica y Química Inorgánica, Facultad de Ciencias, Universidad de Cádiz, Campus Río San Pedro, 11510 Puerto Real, Spain

## Abstract

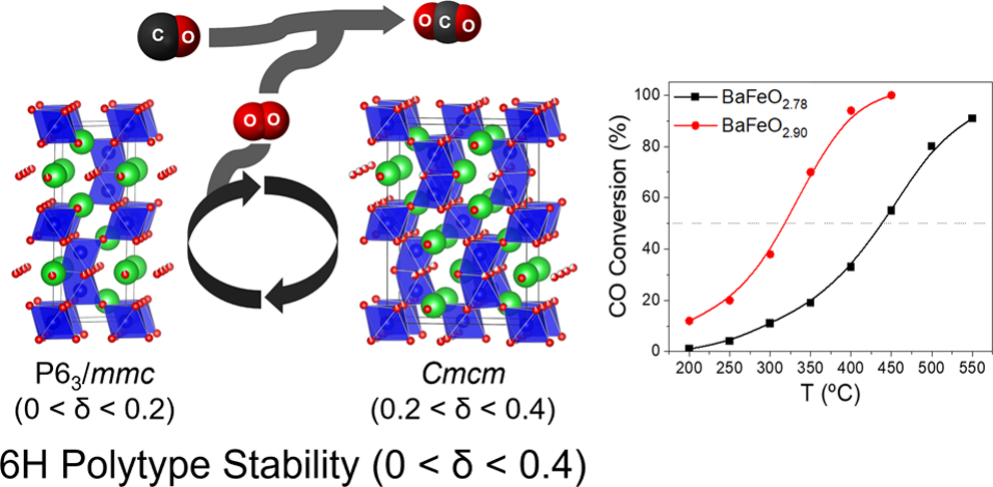

This work is devoted to evaluating the relationship between the oxygen content and catalytic activity in the CO oxidation process of the 6H-type BaFeO_3−δ_ system. Strong evidence is provided about the improvement of catalytic performance with increasing Fe average oxidation state, thus suggesting the involvement of lattice oxygen in the catalytic process. The compositional and structural changes taking place in both the anionic and cationic sublattices of the catalysts during redox cycles have been determined by temperature-resolved neutron diffraction. The obtained results evidence a structural transition from hexagonal (*P*6_3_/*mmc*) to orthorhombic (*Cmcm*) symmetry. This transition is linked to octahedra distortion when the Fe^3+^ concentration exceeds 40% (δ values higher than 0.2). The topotactical character of the redox process is maintained in the δ range 0 < δ < 0.4. This suggests that the cationic framework is only subjected to slight structural modifications during the oxygen exchange process occurring during the catalytic cycle.

## Abstract 2

The topotactic redox reversibility of the 6H-BaFeO_3−δ_ system is studied by time-resolved neutron diffraction and electron microscopy. The influence of the Fe^3+^/Fe^4+^ ratio on CO oxidation is assessed.

## Introduction

AMO_3_ perovskite-related compounds represent probably one of the most studied types of mixed oxides in the field of heterogeneous catalysis. The extraordinary compositional flexibility of the perovskite structure allows tailoring of the oxidation state and the M-cation environment to modulate their catalytic performance. They are considered an alternative to replace noble metals in several catalytic formulations with potential application in a variety of reactions, most of them redox processes. CO oxidation is an example of an important environmental protection process as well as a model reaction to study catalyst mechanisms.

Lanthanide-based perovskites (LaMO_3_, M = first-row transition metal) have been widely studied as catalysts for carbon monoxide oxidation. In particular, there has been a special interest in the influence of manganese, cobalt, and iron as M-cations.^1−4^ Cobalt-based materials seem to exhibit the most promising activities^5,6^ but cobalt is considered a critical raw element by the EU.^7^ Although iron catalysts seem to exhibit poor activity in comparison to other metals, the high availability and low cost of iron precursors have kept lanthanide ferrite materials in the scientific spotlight for many years.

Given the influence of surface area in all heterogeneous processes, the synthesis plays a significant role in catalytic performance. LaFeO_3_ catalytic properties have been improved when synthesized as nanoparticles. The most common synthesis routes to obtain a small particle size of perovskite materials are sol gel and coprecipitation. An alternative route to increase surface area during synthesis is by the generation of pores within the solid. This can be achieved through hard casting, using templates such as mesoporous silica^8^ or poly(methyl methacrylate) nanospheres,^3^ or by nanostructuring of precursors, e.g., from macroporous Fe_2_O_3_.^9^

The effect of partial or total substitution of lanthanum with other cations has also been evaluated. For instance, the catalytic activity for CO oxidation of AFeO_3_ (A = La, Nd, Sm)-related perovskites follows the order Nd > La > Sm, according to Porta et al.^10^ and Ciambelli et al.^11^ Nevertheless, lanthanide elements remain as problematic as cobalt.^7^ As a result, their substitution by other elements has been recently evaluated to obtain more environmentally sustainable catalysts. Partial substitution of lanthanum by alkaline earths such as Sr,^12^ Ca,^13^ or Ba^14^ has been reported to remarkably enhance the catalytic performance of Fe perovskites-related oxides.

However, the potential of precisely tailoring the anionic sublattice, e.g., by controlling the oxygen content or features of the oxygen defect structures, has been scarcely explored until now. In line with this concept and keeping in mind the increasing pressure to replace rare earths by other, noncritical, transition metals, we studied in a previous work the effect of the total substitution of lanthanum by barium in a LaFeO_3_ system on CO oxidation performance.^15^ Complete replacement of La by Ba, by a sol gel method, led to the stabilization of an oxide exhibiting hexagonal symmetry and oxygen vacancies with the composition BaFeO_2.78_. This phase crystallizes in a hexagonal (hcc)_2_-6H polytype with a random distribution of anionic vacancies in both the hexagonal (h) and cubic layers (c). Iron atoms are present in a 3.56 average oxidation state (0.56 Fe^IV^: 0.44 Fe^III^/unit formula). Concerning its catalytic performance, our results showed that BaFeO_2.78_ displays higher catalytic activity than other lanthanide-containing ferrites with a *T*_50%_ (*T*_50%_, also known as light-off temperature, denotes the temperature at which 50% of the reactant, CO in this case, is converted into product, CO_2_) value between 50 and 80 K lower. Temperature-programmed reduction under H_2_ and CO evidenced the involvement of lattice oxygen in CO oxidation, even at low temperatures (ca. 500 K). The removal of lattice oxygen by hydrogen pretreatment significantly decreased the catalytic activity and increased the light-off temperature, thus supporting the involvement of labile oxygen species, as well as the occurrence of an intrafacial reaction mechanism.

From these results, the question arises whether a more oxidized phase, incorporating a larger amount of (Fe^4+^), will exhibit a higher activity. Regarding the availability of the corresponding materials, a few examples in the literature have reported the obtention of fully oxidized BaFeO_3_ but not with the 6H-type structure, which precludes a proper comparison with previous catalytic results. In this regard, a fully oxygenated (hhc)_3_-12R BaFeO_3_ has been obtained by high-pressure synthesis, starting from an oxygen-deficient 6H-BaFeO_3−δ_ as a precursor,^16^ but this method is not suitable for heterogeneous catalysis, as the obtained solids present large particle size. Topotactic oxidation with ozone^17^ appears as a less aggressive procedure. Thus, in an original publication by Kageyama’s group, BaFeO_2.5_ was first obtained by spray pyrolysis. Then, further treatment under ozone led to a cubic 3C-BaFeO_3_ polytype.

In this context, and to isolate the specific influence of the Fe oxidation state, this work explores the synthesis, characterization, and catalytic performance of highly oxidized barium–iron perovskites, obtained by both high and low temperature oxidation treatments, starting from the 6H-BaFeO_2.78_ phase. To this end, oxides with compositions BaFeO_2.96_ (0.92 Fe^IV^: 0.08 Fe^III^/unit formula) and BaFeO_2.90_ (0.80 Fe^IV^: 0.20 Fe^III^/unit formula) have been prepared and characterized to completely assess their oxygen content and microstructural features by using a variety of diffractometric and electron microscopy techniques. In parallel, an in-depth characterization of the BaFeO_3−δ_ redox properties was performed through in situ high-temperature neutron diffraction (HT-ND), selected area electron diffraction (SAED), and high resolution transmission electron microscopy (HRTEM). The influence of oxygen stoichiometry on catalytic CO oxidation activity has been also assessed.

A preprint of this work is available in ChemRxiv.^18^

## Experimental Section

BaFeO_3−δ_ oxides were prepared by the sol gel method. As described in a previous work,^15^ Ba(NO_3_)_2_ (5.23 g, Merck >99%, CAS: 10022-31-8) and Fe(NO_3_)_2_·9H_2_O (8.07g, Merck ≥99.95%, CAS: 7782-61-8) were dissolved in 200 mL of deionized water. Citric acid monohydrate (8.40 g, Merck >99%, CAS: 05949-29-1) and ethylene glycol (45 mL, Merck, CAS: 00107-21-1) were added to the solution under stirring and heating at 120 °C. Then, the solution was further heated until complete evaporation. A dark brown solid was obtained, which was manually ground and then calcined at 350 °C overnight to remove organic residues. The obtained solid was thermally treated at 750 °C for 24 h in air to produce the lowest oxygen content phase, BaFeO_2.78_. BaFeO_2.90_ was obtained by oxidation of the previous sample at 200 °C under an ozone/oxygen flow (ca. 5% O_3_) for 4 h. Ozone was generated in situ by a Pacific Ozone L24 Ozone Generator. BaFeO_2.96_ was obtained from BaFeO_2.78_ at 800 °C under an oxygen flow overnight.

Powder X-ray diffraction (XRD) patterns were collected using Cu Kα monochromatic radiation (λ = 1.54056 Å) at room temperature on a PANalytical X’Pert PRO MPD diffractometer equipped with a germanium(111) primary beam monochromator and X’Celerator fast detector. Diffraction data were analyzed by the Rietveld method^19^ using the FullProf program.^20^

The average cationic composition was determined by energy-dispersive X-ray spectroscopy (EDS). The average oxidation state of iron was determined by a redox titration method, and from this, the oxygen content of the samples is obtained. Samples were dissolved in HCl (6N) with an excess of Mohr salt. The Fe^2+^ ions react with the possible Fe^4+^ present in the sample, leading to Fe^3+^ ions. The amount of remaining Fe^2+^ ions was determined by titration with a 0.1 N K_2_Cr_2_O_7_ solution.

X-ray photoelectron spectroscopy (XPS) analyses were carried out in a SPECS GmbH spectrometer with a PHOIBOS 150 MCD-9 detector, using a nonmonochromatic Mg Kα source (200 W, 12 kV). The data were treated using the CasaXPS software^21^ and the binding energy values were referenced to adventitious carbon.

For electron microscopy characterization, a variety of pieces of equipment were used. The morphological study was carried out on a JEOL JSM 7600F scanning electron microscope operating at an acceleration voltage of 15 kV. Atomic resolution characterization was performed on a JEOL JEM-ARM200cF electron microscope (Cold Emission Gun) operating at 120 kV provided with a spherical aberration corrector in probe (current emission density ∼1.4 × 10^–9^ Ă and probe size ∼ 0.08 nm), a GIF-QuantumER spectrometer, and an Oxford INCA-350 detector. High-angle annular dark-field (HAADF) images were recorded using a nominal camera length of 60 mm and inner and outer collection semiangles of 68 and 280 mrad, respectively. Electron energy-loss spectroscopy (EELS) experiments were acquired using a collection semiangle of 18 mrad and a convergence semiangle of 20.3 mrad. The local iron oxidation state was analyzed from the energy-loss near-edge fine structure (ELNES) of Fe-L_2,3_ signals. EELS spectra were acquired with an energy dispersion of 0.05 eV and a 2 mm spectrometer aperture. The Dual-EELS function of the GIF-QuantumER spectrometer, allowing the simultaneous acquisition of two different energy ranges, was used to record simultaneously the zero-loss peak (0.0001 s exposition time, 10 frames) and Fe-L_2,3_ core-region in order to minimize the uncertainty on the energy shift of the Fe-L_2,3_ edges. In situ TEM annealing experiments were performed with a JEOL JEM 2100HT transmission electron microscope at a temperature of 300 °C by using a Gatan 652 double-tilt heating holder. For in situ annealing TEM and (S)TEM observations, the samples were prepared by crushing in an agate mortar, ultrasonically dispersing the crushed powders in *n*-butanol, and finally transferring them to carbon-coated copper or molybdenum grids.

Neutron powder diffraction (NPD) was carried out on the D2B diffractometer (λ = 1.594 Å) at the Institute Laue Langevin (ILL).^22^ The data were analyzed with the Rietveld^19^ method using the FullProf software package.^20^ Patterns were collected first at room temperature and then at successively higher temperatures in two different atmospheres, vacuum and air. For the vacuum experiment, about 2 g of powder was loaded into a vanadium can and heated using a high-temperature vacuum furnace (ILL-type vacuum furnace). The furnace was pumped throughout the experiment, maintaining a vacuum of about 1 × 10^–4^ bar. In air experiments, the sample was placed in a glass container and heated in an ILL furnace. Sample names and the corresponding chemical compositions of the stabilized phases are shown in Table 3. Further details on the crystal structure of these phases were deposited to the ICSD/Fiz Karlsruhe database with the following reference numbers CSD-2340066–2340075. The reference number of each phase is also gathered in Table 3.

The textural properties of the samples were determined by N_2_ adsorption at −196 °C using an ASAP 2020 analyzer, Micromeritics. Prior to the analysis, the samples were evacuated under vacuum at 200 °C for 2 h.

The catalytic activities of the samples were studied in a U-shaped fixed-bed quartz reactor. A Bruker gas chromatograph (model 450-CG) was used for the analysis. Catalytic activity tests were carried out with step heating from 200 to 550 °C with steps every 50 °C. The temperature was maintained at each step for 30 min at a heating ramp of 5 °C/min. In the tests, 50 mg of the catalyst was mixed with 100 mg of SiC. The catalytic activity test was performed using a reaction mixture consisting of 1 mL/min CO, 0.6 mL/min O_2_, and the remaining He at a total flow rate of 100 mL/min. The space velocity was 120,000 cm^3^/hg.

## Results and Discussion

As we have indicated above, by using the sol gel synthesis method, with the procedure described in the Experimental Section, a 6H-BaFeO_2.78_ phase is obtained. The choice of the sol gel method is not by chance. First, it is one of the few methods in which the oxygen content can be controlled while being reproducible. Moreover, the particle size of the as-prepared products can be kept within the nanometer range in contrast to the solid-state reaction. Two additional oxidation processes were applied to the BaFeO_2.78_ phase to obtain BaFeO_2.90_ and BaFeO_2.96_.

The Ba/Fe = 1:1 ratio in all of the samples was confirmed by EDS. The average chemical composition of the prepared samples (BaFeO_2.78_, BaFeO_2.96_, and BaFeO_2.90_) was obtained from the Fe^4+^/Fe^3+^ ratio determined by chemical analysis as described in the Experimental Section [Table S1 in Supporting Information (SI)]. Surface composition is also evaluated through XPS. The presence of surface Fe^4+^ in different ratios is confirmed in every sample. A detailed analysis of the collected spectra (Figure S1) is included in the Supporting Information.

The (hcc)_2_-6H structure for all three compositions was verified by X-ray diffraction and electron microscopy. The X-ray patterns can be indexed on the basis of a hexagonal unit cell with space group *P*6_3_/*mmc*; the lattice parameters (listed in Table S1) decrease with increasing Fe(IV) content, as expected from the smaller size of Fe(IV), in comparison with Fe(III).

**Figure 1:**
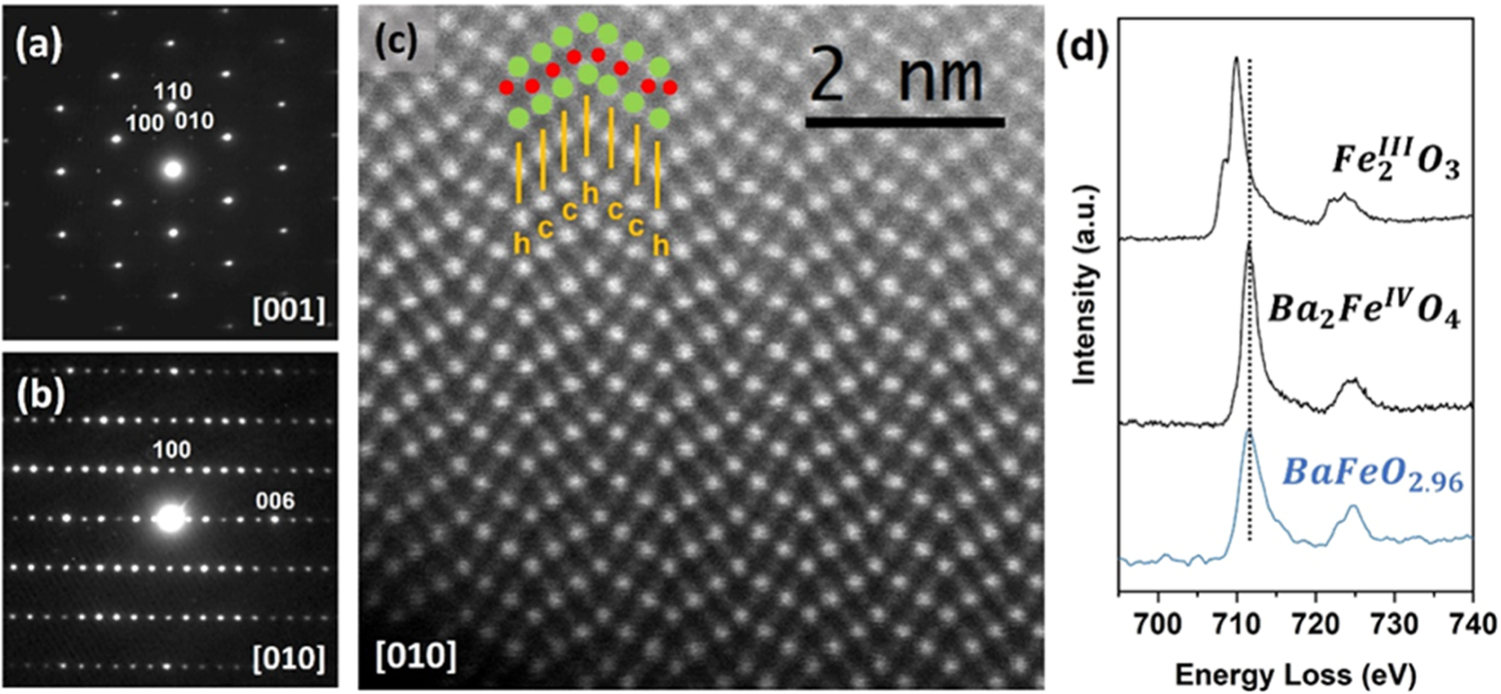
SAED patterns of a BaFeO_2.96_ crystal along the (a) [001] and (b) [010] zone axes. (c) Atomically resolved HAADF along [010] showing the 6H packing (hcchcc) along the *c* axis. Green and red dots indicate Ba and Fe positions, respectively. (d) EELS Fe-L_2,3_ edges of references with Fe(III) and Fe(IV) oxidation states (black lines); BaFeO_2.96_ sample (blue).

The 6H polytype was further confirmed using atomic resolution scanning transmission electron microscopy (STEM). Selected area electron diffraction (SAED) patterns along [001] and [010] of a representative BaFeO_2.96_ crystal are shown in Figure 1a,b, respectively. The reflection conditions are consistent with the 6H-hexagonal cell, and the sharp maxima observed indicate a lack of significant concentration of structural defects. The high-angle annular dark-field (HAADF) image along the [010] direction is shown in Figure 1c. The Ba atomic columns (*Z* = 56) can be identified as the brightest contrast, while Fe atomic columns (*Z* = 26) appear with less bright intensity. The 6H stacking sequence cchcch can be clearly identified along the *c* axis (marked by yellow lines). Figure 1d shows the corresponding EELS signal of the Fe-L_2,3_ edge. High energy resolution EELS has been demonstrated to be highly sensitive to local variations of the transition metal oxidation state, and a clear variation in the shape and the energy position is observed as a function of Fe oxidation state for Fe_2_^(III)^O_3_ and Ba_2_Fe^(IV)^O_4_ oxides. The Fe-L_2,3_ edge corresponding to the BaFeO_2.96_ material shows a I(L_3_)/I(L_2_) ratio as well as energy-loss positions of the L_3_ and L_2_ peaks very close to those of Ba_2_FeO_4_, confirming the Fe(IV) oxidation state for the 6H-BaFeO_2.96_ oxide.

The slightly larger width of both peaks, in comparison with that of the reference Fe(IV) signal, is related to the presence of the small, 8%, amount of Fe(III) expected for the BaFeO_2.96_ composition. Notice that clear differences are observed in both the energy loss and the shape of the Fe-L_2,3_ signal when a comparison is made with the signal previously reported for BaFeO_2.78_^15^ (see SI, Figure S2), with a much larger, 44%, content of Fe(III).

Our previous work on BaFeO_2.78_^15^ in situ electron diffraction and XRD data evidenced the structural changes involved in the reduction process of this sample. This study explored the reduction pathway of the 6H polytype from the most oxidized form, BaFeO_2.96,_ to the most reduced one, BaFeO_2.63_. To better understand the evolution of the structure and the oxygen content variation during this redox process, a temperature-resolved neutron diffraction and electron microscopy study of BaFeO_2.96_ oxide after treatment at different temperatures and gas environments (vacuum and air) is now performed.

### Neutron Diffraction Study

**Figure 2:**
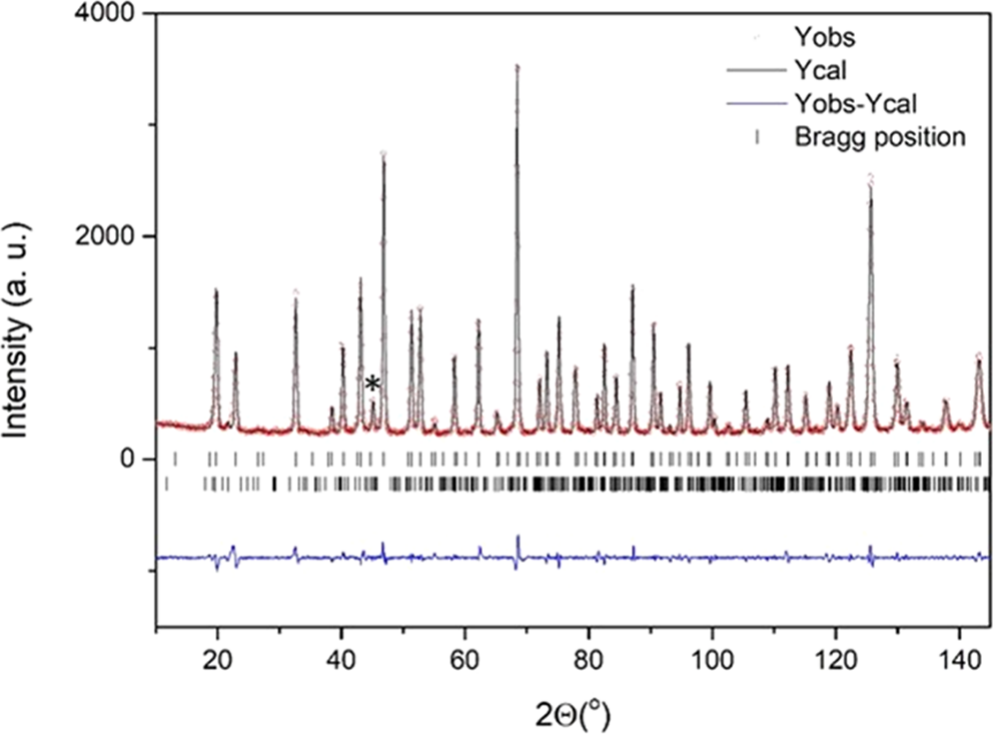
Final Rietveld refinement of the NPD data measured at RT for the H-BaFeO_2.96_ pristine oxide. The observed (red circles) and calculated patterns (continuous black line), and difference curves (continuous blue line) are shown. Upper tick marks correspond to the main 6H-BaFeO_2.96_ phase and lower ticks correspond to BaFe_2_O_4_ added as a secondary phase (its most intense reflection is marked with an asterisk in figure).

**Figure 3:**
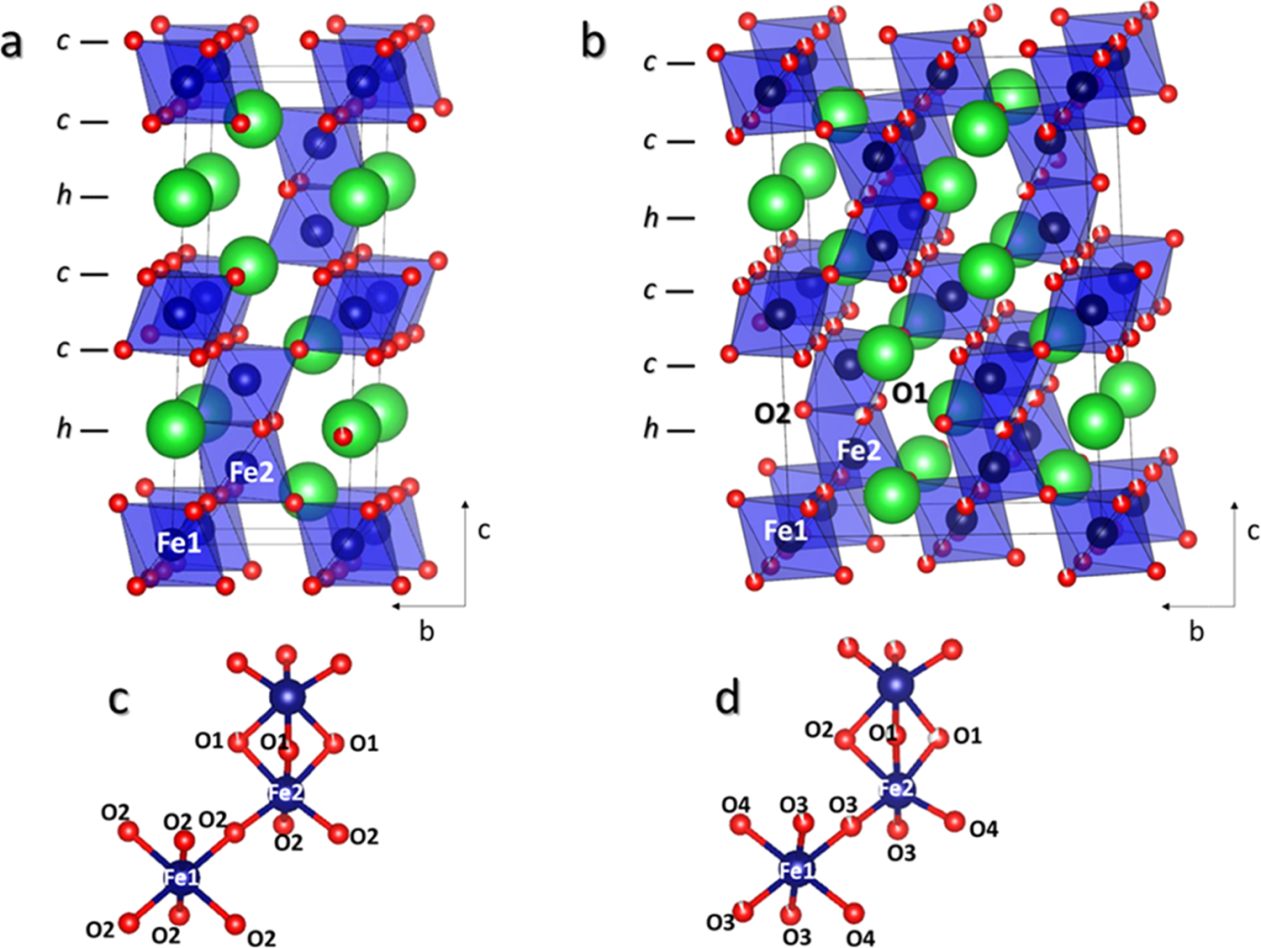
Schematic representation of the crystal structures of (a) hexagonal 6H-BaFeO_2.96_ and (b) orthorhombic O-BaFeO_2.63_. Corresponding oxygen polyhedral coordinations for Fe1 and Fe2 are shown in panels (c) and (d) for 6H-hexagonal and orthorhombic phases, respectively.

The crystal structure analysis with powder neutron diffraction at room temperature (RT1) confirms that BaFeO_2.96_ adopts the 6H-type with hexagonal symmetry and S.G. *P*6_3_/*mmc*. The structural parameters of BaFeO_2.78_^15,23^ were taken as starting values for refinement. The final results are listed in Figure 2. Note that a small amount (less than 4% mass) of BaFe_2_O_4_ is detected by NPD. The oxygen occupancies were refined and the stoichiometry BaFeO_2.965(7)_ was determined for the high-temperature oxidized sample which perfectly matched the chemical analysis result. The small fraction of oxygen vacancies is located in the hexagonal layers (O1). The refined structural data are given in Table 1, while Table 2 shows the bond distances of this phase compared to those of the RT2 and RT3 samples (later commented in the manuscript). A depiction of the crystal structure is shown in Figure 3. As can be seen in Table 2, Fe1 octahedron (isolated corner sharing one) is symmetrical, presenting six identical Fe–O distances (2.025 Å) and O–Fe–O bond angles of 90 and 180°, whereas [Fe2O_6_] octahedra (face-sharing ones) are slightly distorted with three longer and three shorter distances. This distortion is due to the displacement of Fe2 from the octahedron center toward the adjacent cubic layers to reduce electrostatic repulsions between the metal ions located in the face-shared dimers. This is a general feature in hexagonal polytypes.

**Table 1:** Crystallographic Parameters Refined from ND Data for the RT1 (6H-BaFeO_2.96_) Sample (CSD: 2340066)

RT1 6H-BaFeO_2.96_	*x*	*y*	*z*	Biso (Å^2^)	Occ
Ba1	0	0	0.25	0.35(6)	1
Ba2	0.3333	0.6666	0.59135(18)	0.38(6)	1
Fe1	0	0	0	0.10(3)	1
Fe2	0.3333	0.6666	0.15489(11)	0.79(3)	1
O1	0.4853(5)	0.9702(9)	0.25	0.81(5)	0.965(7)
O2	0.1687(5)	0.3372(10)	0.41587(10)	1.12(3)	1
*a* = 5.66373(4), *c* = 13.87814(15) Å hexagonal symmetry (*P*6_3_/*mmc*)					
*R*_B_ = 1.77, χ^2^ = 4.84; *R*_p_ = 2.83; *R*_exp_ = 1.83					

**Table 2:** Bond Distances and Octahedral Distortion^a^ for RT1 (6H-BaFeO_2.96_), RT2-Vacuum (O-BaFeO_2.63_), and RT3-Air (6H-BaFeO_2.86_) Samples (CSD: 2340066, 2340070, and 2340073, Respectively)

6H-BaFeO_2.96_ (RT1)		O-BaFeO_2.63_ (RT2-vacuum)		6H-BaFeO_2.86_ (RT3-air)	
Fe1–O octahedra		Fe1–O octahedra		Fe1–O2 octahedra	
Fe1–O2	2.025(3) × 6	Fe1–O3	2.052(6) × 4	Fe1–O2	2.040(4) × 6
		Fe1–O4	2.040(9) × 2		
		average distance	2.047(3)		
distortion: 0		distortion: 5.7 × 10^–5^		distortion: 7 × 10^–6^	
Fe2–O octahedra		Fe2–O octahedra		Fe2–O octahedra	
Fe2–O1	1.99(3)	Fe2–O1	1.971(7) × 2	Fe2–O1	2.030(3) × 3
Fe2–O2	1.891(3)	Fe2–O2	2.136(11)	Fe2–O2	1.869(4) × 3
average distance	1.9401(3)	Fe2–O3	1.882(7) × 2	average distance	1.9498(14)
		Fe2–O4	1.821(11)		
		average distance	1.944(4)		
distortion: 6.523 × 10^–4^		distortion: 2.6882 × 10^–3^		distortion: 1.716 × 10^–3^	

a Octahedra distortion is calculated from , where *d*(Fe–O*i*) is the interatomic Fe–O(*i*) (*i* = 1–6) distances while ⟨*d*(Fe–O)⟩ corresponds to the average distance Fe–O.

To analyze the reduction process, the BaFeO_2.96_ sample was heated from RT (sample RT1) to 550 °C and cooled to RT under a vacuum (sample RT2). Above this temperature, the 6H stacking breaks down due to the random insertion of cubic layers, resulting in an irreversible structural change preventing the analysis of the reduction and oxidation process. Therefore, the highest temperature for the experiment was fixed at 550 °C. The oxidation process was carried out in air. Under this atmosphere, the RT2 sample was heated again to 450 °C and then cooled to RT (sample RT3). The temperatures at which diffraction patterns are collected in both reduction and oxidation processes are gathered in Table 3.

**Table 3:** Temperatures of ND Data Collection During Reduction and Oxidation Steps^a^

	reduction process (vacuum)				
	heating			cooling	
temperature (°C)	RT1	350	550	300	RT2-vacuum
composition	6H-BaFeO_2.965(7)_	O-BaFeO_2.669(17)_	O-BaFeO_2.651(16)_	O-BaFeO_2.652(17)_	O-BaFeO_2.629(16)_
CSD *N*°	2340066	2340067	2340068	2340069	2340070
average Fe oxidation state	3.92	3.34	3.30	3.30	3.26

	oxidation process (air)				
	heating			cooling	
temperature (°C)	RT2-vacuum	200	450	300	RT3-air
composition	O-BaFeO_2.647(15)_	O-BaFeO_2.690(16)_	O-BaFeO_2.71(2)_	H-BaFeO_2.892(5)_	H-BaFeO_2.864(6)_
CSD *N*°	2340071	2340072	2340073	2340074	2340075
average Fe oxidation state	3.29	3.38	3.42	3.82	3.76

a H and O refer to hexagonal and orthorhombic symmetry, respectively.

Neutron diffraction patterns of reduced samples (measured in vacuum at 350 and 550 °C, during the heating process, and at 300 °C and RT2 during the cooling one) do not fit the hexagonal *P*6_3_/*mmc* symmetry since some diffraction maxima, in the 2θ range of 81–91°, are split into two. Those maxima are highlighted in Figure S3 (SI), evidencing the differences between the starting hexagonal BaFeO_2.96_ oxide and the RT2 sample (cooling down RT in a vacuum). According to that, the reduction process of the hexagonal BaFeO_2.96_ sample seems to be accompanied by a slight structural distortion which changes the unit cell symmetry from hexagonal to the related orthorhombic one (*Cmcm*). These symmetry-lowering distortions are quite common when two cations of very different sizes occupy the two octahedral sites of the 6H-structure.^24−26^

**Figure 4:**
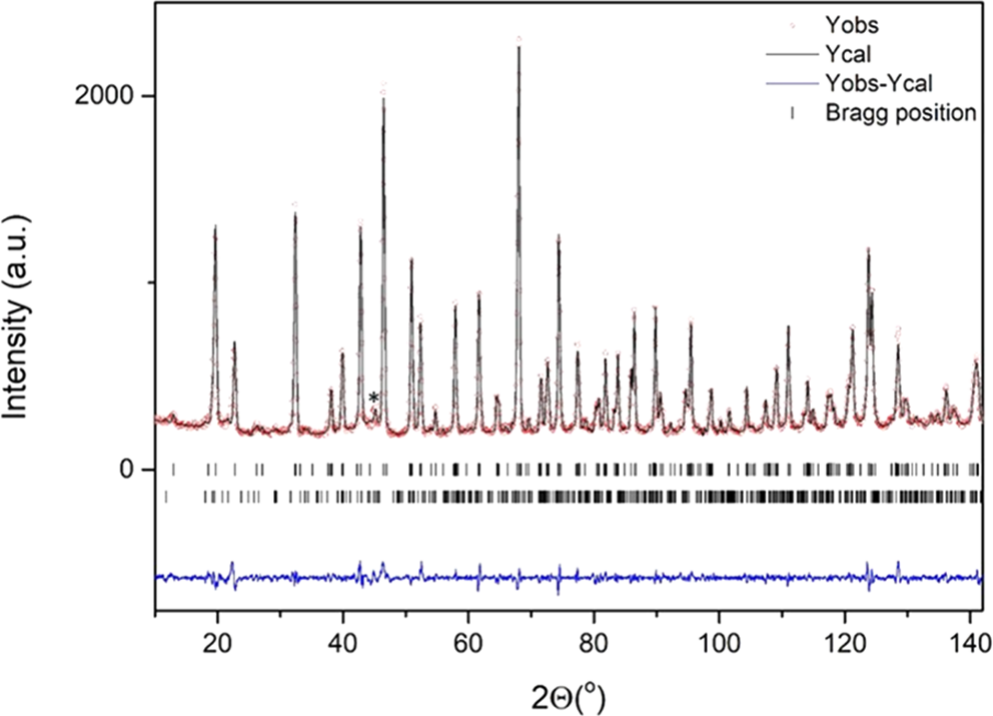
Final Rietveld refinement of the NPD data measured at RT for O-BaFeO_2.629_ (RT2-vacuum sample). Observed (red circles) and calculated patterns (continuous black line and difference curves, and continuous blue line) are shown. Lower ticks correspond to BaFe_2_O_4_ appearing as minority phase (the most intense reflection is marked with an asterisk).

The hexagonal *P*6_3_/*mmc* and the orthorhombic *Cmcm* (maximal nonisomorphous subgroup) cells are related by the transformation matrix (1), giving the following relationships between the hexagonal and orthorhombic unit cell parameters: *a*_ort_ = *a*_H_; *b*_ort_ = √3*a*_H_; *c*_ort_ = *c*_H_. The atomic coordinates in this new orthorhombic unit cell are obtained by using the tools provided in the Bilbao Crystallographic Server.^27−29^
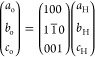
1Therefore, the patterns of the vacuum-reduced samples (named hereafter the O-BaFeO_3−δ_ samples) were fitted to the orthorhombic *Cmcm* space group. The graphic result of the refinement for the RT2-vacuum sample is depicted in Figure 4 and the corresponding structure model is shown in Figure 3b. The crystallographic parameters and bond distances are listed in Tables 4 and 2, respectively. The crystallographic parameters refined from the NDP data at 350 °C (O-BaFeO_2.669_) and 550 °C (O-BaFeO_2.651_) are collected in Table S2 in SI. Refinement shows that all orthorhombic samples have a lower oxygen content than the hexagonal ones, although with a higher proportion in the former. The oxygen vacancies are in the O1 and O3 sites, then distributed through both hexagonal and cubic layers. The anionic composition from the NDP refinements and calculated Fe average oxidation state for each temperature are gathered in Table 3.

**Table 4:** Crystallographic Parameters Refined from the NDP Data for the Samples of the O-BaFeO_2.63_ (RT2-Vacuum) and H-BaFeO_2.86_ (RT3-Air) (CSD: 2340070 and 2340074)

O-BaFeO_2.629(16)_ RT2-vacuum	*x*	*y*	*z*	Biso	Occ
Ba1	0	0.0001(1)	0.25	1.07(3)	1
Ba2	0.5	0.8317(11)	0.58799(18)	1.07(3)	1
Fe1	0	0	0	0.10(3)	1
Fe2	0.5	0.8279(6)	0.15017(9)	0.88(2)	1
O1	0.2764(18)	0.7722(11)	0.25	1.25(7)	0.327(4)
O2	0	0.4915(13)	0.25	1.25(7)	1
O3	0.7504(12)	0.9110(8)	0.4151(4)	1.25(7)	0.895(7)
O4	0.171(1)	0.4183(7)		1.25(7)	1
*a* = 5.669541(17), *b* = 9.8640(3); *c* = 14.02405(11) Å orthorhombic symmetry (*Cmmc*)					
*R*_B_ = 2.72, χ^2^ = 2.27; *R*_p_ = 3.24; *R*_exp_ = 2.83					

H-BaFeO_2.864(6)_ RT3	*x*	*y*	*z*	Biso	Occ
Ba1	0	0	0.25	*	1
Ba2	0.3333	0.6666	0.5918(3)	*	1
Fe1	0	0	0	*	1
Fe2	0.3333	0.6666	0.15138(15)	*	1
O1	0.4850(6)	0.9699(13)	0.25	*	0.865(6)
O2	0.1679(7)	0.33959(14)	0.41523(15)	*	1
*B overall = 1.486(14) Å^2^					
*a* = 5.68455(3); *c* = 13.95676(13) Å hexagonal symmetry (*P*6_3_/*mmc*)					
*R*_B_ = 2.72, χ^2^ = 3.20; *R*_p_ = 2.67; *R*_exp_ = 2.01					

The most reduced sample, attained after cooling to room temperature in vacuum (RT2-vacuum), O-BaFeO_2.629(16)_, was taken as the starting sample for the oxidation process. This process was carried out by heating the sample to 450 °C and then cooling to RT in air (RT3-air). In this temperature range, the redox process is reversible so that during air cooling, the hexagonal symmetry is recovered from 300 °C, attaining a hexagonal symmetry phase with H-BaFeO_2.86_ composition at RT. The refined crystallographic parameters and bond distance values for the RT3-air sample are given in Tables 3 and 2, respectively. Refinement results from ND data collected at intermediate temperatures during both heating and cooling processes are gathered in the SI (Tables S3 and S4)

It should be noted that, as indicated in Table 3, all of the orthorhombic phases, O-BaFeO_2.71_, O-BaFeO_2.67_, O-BaFeO_2.65_, and O-BaFeO_2.63_, have an oxygen content very close to the previously reported BaFeO_2.67_ oxide^30^ described as a hexagonal phase, *P*6_3_/*mmc* symmetry, from the refinement of X-ray data. Neutron diffraction has allowed us both to accurately determine the oxygen composition of each phase and to elucidate the actual orthorhombic symmetry of the BaFeO_3−δ_ phases in a δ range close to 0.37 ≥ δ ≥ 0.3. Furthermore, revisiting our previously published ND data on the BaFeO_2.78_ oxide,^15^ we were able to find the aforementioned splitting maxima associated with the orthorhombic distortion. A better fit with orthorhombic symmetry is provided in the Supporting Information (Figure S4 and Table S5), which widens the stability range of orthorhombic symmetry to near BaFeO_2.63–2.78_.

The reduction process from H-BaFeO_2.96_ to O-BaFeO_2.63_ is accompanied by an increase in the lattice parameters. The variation of the lattice parameters (referred to as the hexagonal cell) measured at RT shows an increase in the lattice parameters as the oxygen content of the samples decreases, Figure S5. This variation is related to the oxidation state of iron, from the smaller tetravalent state present mainly in BaFe^3.92+^O_2.96_ to the larger trivalent state in BaFe^3.26+^O_2.63_.

In both hexagonal and orthorhombic phases, Fe is in octahedral coordination, either sharing faces to form a dimer (Fe2) or sharing corners with dimers (Fe1). Table 2 collects the Fe–O distances corresponding to H-BaFeO_2.96_, H-BaFeO_2.86_, and O-BaFeO_2.63_ phases. [Fe1O_6_] is the largest and most regular octahedron. The Fe1–O distances increase significantly with increasing oxygen vacancy content (the mean dFe–O distance values are 2.025 in H-BaFe_2.96_ and about 2.049 Å in the reduced H-BaFeO_2.86_ and O-BaFeO_2.63_ phases). The size of the [Fe2O_6_] octahedron, smaller than the previous one, is similar in the three samples (average dFe2-O close to 1.94 Å) but the degree of distortion increases with the oxygen vacancy content (see Table 2).

These structural features suggest a possible distribution of Fe(III) and (IV) in both types of octahedra based on the different sizes of the two ions. The larger cation, Fe(III), would be mainly located in the larger octahedra [Fe1O_6_], while the smaller one, Fe(IV), will occupy the Fe2 position. Considering that the relation between Fe1 and Fe2 sites is 1:2, the limit of this ordered distribution would be 1Fe(III):2Fe(IV) which corresponds to a BaFeO_2.83_ composition. This could explain the increase in the Fe1–O2 distance, as the Fe(III) content increases from 2.96 to 2.86 compositions, while Fe2–O remains almost constant. When the amount of Fe(III) is higher than 33%, a fraction of this cation must also occupy the [Fe2O_6_] octahedra, inducing an increase in the average Fe–O distance and in the octahedra degree of distortion, as can be seen in the O-BaFeO_2.63_ phase which reaches a value of 2.6882 × 10^–3^ (Table 2).

These symmetry-lowering distortions are quite usual where two cations of very different size occupy the two different octahedral sites of the 6H-structure.^25,26^ In our case, the higher degree of distortion of the octahedra as the Fe(III) content increases could be at the origin of the observed transition phase from hexagonal to orthorhombic. Furthermore, following the above reasoning, we can establish that the hexagonal phase is stable if the Fe2 site is mostly occupied by Fe(IV), i.e., in the compositional range from BaFeO_3_ to near BaFeO_2.83_. For lower oxygen contents, the orthorhombic phase is stabilized.

### Microstructural Study: SAED and HRTEM

The microstructural changes in the BaFeO_2.96_ sample during the reduction and oxidation steps were monitored by using a heating stage to record diffraction and imaging transmission electron microscopy data as a function of temperature.

To mimic the temperature conditions during the neutron diffraction experiment, the sample was initially heated in the microscope up to 550 °C. Under these conditions, the crystals suffer a structural transition leading to the irreversible loss of the 6H packing (detailed in Figure S6), which suggests that the sample reached a highly reduced state. This discrepancy with the neutron diffraction experiments can be explained by the additional reducing conditions associated with electron microscopy (high vacuum and electron beam irradiation).

**Figure 5:**
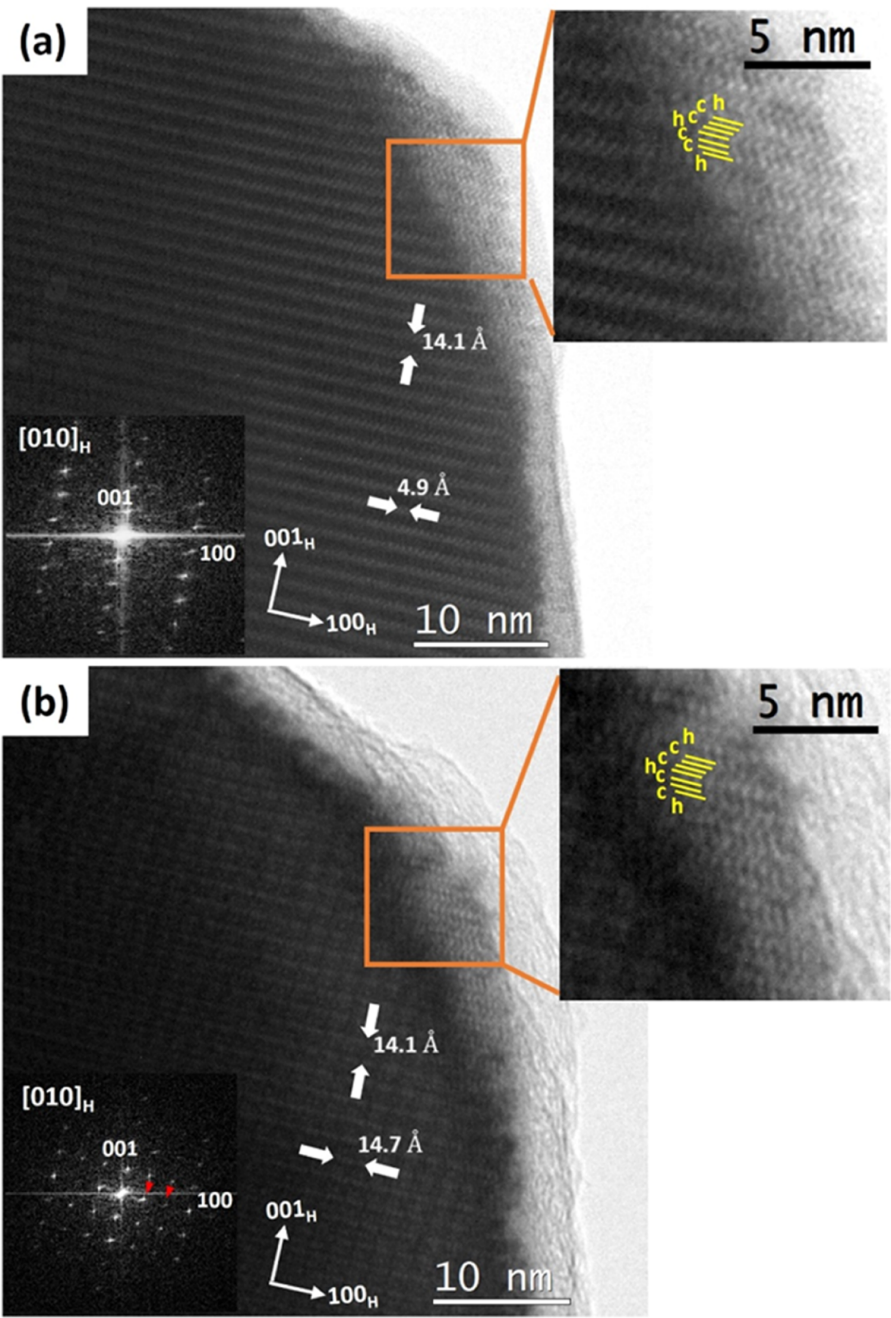
HRTEM images along [010] of the H-BaFeO_2.96_ sample recorded during the in situ heating experiment under vacuum at (a) 25 °C (pristine sample) and (b) 300 °C. A 14.1 Å distance can be measured along 001_H_ in both images. An extra order along 100_H_ is observed with a periodicity of 14.7 Å which corresponds to 3*d*_(100)H_. Corresponding fast Fourier transform (FFT) are shown as the inset. The enlarged image of the thinnest regions of the crystals (marked in orange) evidence the cchcch sequence of the 6H polytype.

To get information about the redox process in hexagonal BaFeO_2.96_, the temperature for the electron microscopy experiment was optimized to 300 °C to prevent an irreversible transition to a nonhexagonal-type phase. At 300 °C, the presence of extra superstructure maxima along the (010)* and (110)* and equivalent reciprocal 6H directions is observed (see Figure S7c,d), suggesting that the sample is reduced and the oxygen vacancies accommodate in the *ab* plane of the structure, as previously described for the BaFeO_2.78_ composition.^15^ The analysis of the contrasts of the HRTEM images recorded along [010]_H_ projection (see Figure 5) evidence that the formation of oxygen vacancies in BaFeO_2.96_ does not break the -cchcch- packing sequence along the *c* axis of the 6H polytype. It is worth mentioning that, although the hexagonal packing is kept, marked differences in the intensity of the contrasts along the *c* axis are observed when comparing the image recorded at room temperature (pristine sample) with the one recorded at 300 °C; this suggests that the oxygen vacancy ordering involves certain displacement of the cationic sublattices.

When the sample is cooled down to room temperature under the vacuum conditions of the electron microscope, such extra structural order vanishes, and the acquired diffraction patterns shown in Figure S7e,f and the corresponding images in Figure S8 resemble those recorded from the pristine sample. Since the reducing conditions of the electron microscope do not allow the reoxidation of the sample, we could assume that the oxygen content of the cooled sample should be similar to the one at 300 °C. Therefore, on cooling, the oxygen vacancies may become randomly distributed within the BaO_3–*x*_ layers, while the cations occupy the positions of the initial 6H scaffold.

To determine the oxygen content as well as the complete structural description of the aforementioned 3-fold superstructure phase detected by in situ electron microscopy, several neutron diffraction experiments were attempted. Unfortunately, the elusive experimental conditions to obtain this metastable phase were not found. Nevertheless, considering the 6H packing is kept during the heating and cooling process, an oxygen content between 0 < δ < 0.4 can be suggested (see discussion of tentative structural model in SI), highlighting once more the topotacticity of this system. This fact shows again the wide range of anionic deficiency that the BaFeO_3−δ_ system can accommodate while preserving the hexagonal structure, a fact that must influence the reversibility of the redox process involved in their catalytic activity.

### Catalytic Activity

As indicated in the introduction, ABO_3_ perovskite-type oxides have been widely studied as catalysts for the CO oxidation reaction, mainly due to their high activity and stability. The catalytic performance of these materials depends on aspects such as the nature of the A and B sites, the crystalline structure, and the surface area, among others. In a previous work, we reported that BaFeO_2.78_ with hexagonal symmetry exhibited higher activity than other lanthanide-containing ferrites. We demonstrated the involvement of labile oxygen species in the CO oxidation mechanism, thus suggesting the possibility of improving the catalytic performance by using samples with a higher degree of oxidation. The main challenge in confirming this hypothesis is the possibility of obtaining samples more oxidized than BaFeO_2.78_ while preserving relevant catalytic properties such as the crystal structure and surface area. This objective was achieved by using a low temperature oxidation method to obtain the BaFeO_2.90_ oxide from the BaFeO_2.78_ phase.

**Figure 6:**
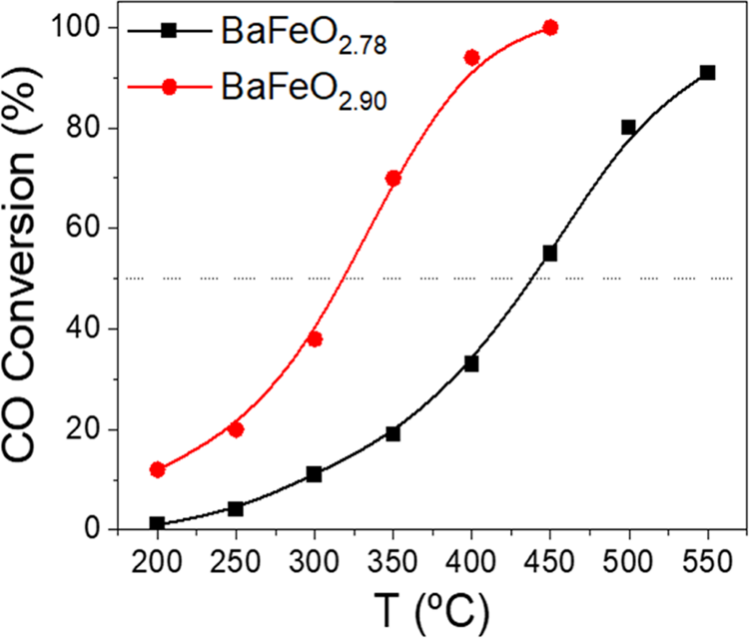
Light-off curves of CO conversion for the CO oxidation reaction over BaFeO_2.78_ and BaFeO_2.90_ perovskites.

The CO conversion values obtained for BaFeO_2.90_ and BaFeO_2.78_ oxides as a function of the reaction temperature are shown in Figure 6. BaFeO_2.96_ catalytic results (Figure S12) are not included in the main text, since oxidation at high temperatures allowed us to reach a higher iron oxidation state at the expense of lower specific surface area (<1 m^2^/g), rendering them hardly comparable. On the other hand, the other two samples exhibit similar morphological and textural properties as revealed by SEM (Figure S10) and physisorption data (Table S6 and Figure S11).

BaFeO_2.90_ exhibits better performance over the whole temperature range tested, achieving conversion values significantly higher than those of BaFeO_2.78_ even at low temperatures. The superior behavior of BaFeO_2.90_ is also evident from the values obtained for the light-off temperature which is almost 120 °C lower for BaFeO_2.90_ (320 and 440 °C for BaFeO_2.90_ and BaFeO_2.78_, respectively). According to the previous characterization, the above results suggest a clear influence of the oxidation state of iron on the CO conversion capacity of the perovskite, which increases as the Fe^4+^ content increases or, in other words, increases with the Fe oxidation degree. It is commonly accepted that the catalytic activity of perovskite oxides is linked, among other factors, to the coexistence of different oxidation states of transition metal ions and the amount of oxygen vacancies. Assuming the participation of oxygen vacancies in the adsorption and dissociation of the oxygen molecule on the surface and in the subsequent incorporation of the oxygen into the oxide lattice, our results point out that the Fe^4+^/Fe^3+^ ratio would be the key parameter to explain the catalytic activities shown by the BaFeO_3−δ_ oxides here investigated. Accordingly, we may propose a mechanism for CO oxidation on these materials based on a redox cycle, in which iron is initially reduced, by providing the necessary oxygen for CO oxidation, and subsequently undergoes reoxidation through interaction with gaseous oxygen. This mechanism is allowed by the remarkable reversibility of the anionic lattice to release and incorporate oxygen over a very large compositional range (0 < δ < 0.40) while keeping the main structural features of the 6H polytype.

The predominance of the Fe^4+^/Fe^3+^ ratio over the oxygen vacancy concentration as the determining factor of the catalytic activity was also pointed out by Barbero et al.^31^ in their investigation of the catalytic performance of LaFeO_3_ perovskites doped with varying amounts of Ca for ethanol oxidation. It is noteworthy, however, that these authors reported an inverse order of influence for both aspects (number of vacancies > Fe^4+^/Fe^3+^ ratio) in the case of the oxidation of another type of molecule, such as propane.

The higher performance observed for the oxide with a higher Fe^4+^ content is also in agreement with other results previously reported. Thus, Tascón et al.^32^ investigated the CO oxidation behavior of a series of LaBO_3_ oxides (B: first-row transition metal) and found a correlation between the catalytic activity and the electronic configuration of the transition metal at the B position. Maxima were observed for the d^4^ (Mn^3+^, Fe^4+^) and d^6^ (Co^3+^) configurations, while minima were observed for the d^5^ (Fe^3+^) configuration. These authors conclude that the local surface symmetry strongly influences the adsorption and catalytic properties of perovskites. In this sense, it is commonly assumed that the e_g_ orbitals of the B cation present optimal geometry (vertically oriented) for interacting with the molecular orbitals of the CO and the O_2_ molecules. Moreover, the binding strength of adsorbates depends on the occupancy of the e_g_ orbital, being optimal (neither too strongly nor too weakly) for an e_g_ filling of 1, as in the case of Fe^4+^.^33^

In summary, the results obtained allow us to conclude that the two BaFeO_3−δ_ oxides with the same structure and quite close textural characteristics, differing only in the degree of oxidation of Fe, show clearly different catalytic behavior in a model oxidation process such as the transformation of CO into CO_2_.

## Conclusions

6H-BaFeO_2.78_ has been synthesized by the sol gel method as starting material to get higher oxidized compositions. Following two different approaches, high-temperature calcination under oxygen and low-temperature treatment with ozone, BaFeO_2.96_ and BaFeO_2.90_, respectively,_,_ have been successfully prepared.

The redox behavior of the most oxidized phase, BaFeO_2.96_, with 6H-structure (*P*6_3_/*mmc* symmetry) has been monitored by temperature-resolved neutron diffraction under vacuum (reduction) and air (oxidation) atmospheres in the temperature range between 25 and 550 °C. These experiments allowed us to precisely quantify and locate the generated oxygen vacancies under vacuum within the 6H-framework. A lowering-symmetry phase transition from hexagonal to orthorhombic *Cmcm* has been identified during the reduction process when the oxygen vacancy content reaches 22%, i.e., for a BaFeO_2.78_ composition. Reoxidation of the most reduced sample, O-BaFeO_2.63_, occurs by heating to 400 °C and cooling in air, via a topotactic pathway, recovering the hexagonal symmetry and displaying the redox reversibility of the Ba–Fe–O system.

The influence of the initial vacancy concentration and iron oxidation state on the catalytic response of the Ba–Fe–O system in the CO oxidation process was studied by comparing the BaFeO_2.78_ and BaFeO_2.90_ samples with similar textural characteristics. A direct comparison of the light-off temperatures shows that BaFeO_2.90_ shows higher CO conversion, supporting the Fe^4+/^Fe^3+^ ratio as the predominant factor of the catalytic activity over the oxygen vacancies concentration. The obtained results show the Ba–Fe–O system, in particular the BaFeO_2.90_ 6H phase, as an outstanding noble-metal and lanthanide-free catalyst for CO conversion.

After the preparation of this manuscript and before sending it for publication, our attention has been drawn to an article about the oxygen release and incorporation in BaFeO_3_ polymorphs (Watanabe et al).^34^ A fully oxygenated 6H-BaFeO_3_ sample has been prepared by using high-pressure (8 GPa) and high-temperature (1000 °C) conditions with KClO_4_ as the oxidizing agent. These authors have described that sample 6H loses weight when heated in air to 600 °C, obtaining a composition close to BaFeO_2.8_. The temperature-dependent synchrotron X-ray diffraction in situ shows that the 6H-type is essentially the same keeping the *P*6_3_/*mmc* hexagonal symmetry from room temperature to 600 °C.

In this sense, we want to highlight that our redox study on BaFeO_3−δ_ samples with small particle size, heating up to 550 °C and working in different atmospheres, vacuum and air, extends the anionic compositional range in which the basic 6H-structure remains to be δ = 0–0.37, which is significantly wider than that reported by these authors allowing us to evidence, for the first time, the existence of a lowering-symmetry phase transition from hexagonal to orthorhombic (*Cmcm*) symmetry that takes place when the oxygen composition is less than about 2.8 (δ close to 0.2) up to 2.63 (δ close to 0.37).

## Supporting Information Available

Additional structural, chemical, and catalytic characterization (PDF)
