# Supplementary material for: Exploring Reversible Redox Behavior in the 6H-BaFeO3−δ (0 < δ < 0.4) System: Impact of Fe3+/Fe4+ Ratio on CO Oxidation
Source: Inorg Chem. 2024 Apr 29;63(19):8908–18. doi: 10.1021/acs.inorgchem.4c00917 (PMC11094785; doi:10.1021/acs.inorgchem.4c00917)
Supplement: Supplementary file 1 — ic4c00917_si_001.pdf [file ic4c00917_si_001.pdf]

# Supporting Information

## *Exploring reversible redox behavior in the 6H-$BaFeO_{3-\delta}$ ($0 < \delta < 0.4$) system. Impact of $Fe^{3+}/Fe^{4+}$ ratio on CO oxidation.*

*D. Gutiérrez-Martín[a], A. Varela[a], M. Hernando[a], A. Torres-Pardo[a], E. Matesanz[b], I. Gómez-Recio[a], J.M. González-Calbet[a], M.T. Fernández-Díaz[c], J.J. Calvino[d], M.A. Cauqui[d], M.P. Yeste[d], and M. Parras[a]*.*

***mparras@ucm.es***

[a] Departamento de Química Inorgánica, Facultad de Ciencias Químicas, Universidad Complutense, 28040 Madrid, Spain.

[b] Unidad de Difracción de Rayos X. Centro de Asistencia a la Investigación de Técnicas Químicas, Universidad Complutense de Madrid, 28040 Madrid, Spain.

[c] Institut Laue-Langevin,38042 Grenoble cedex 9, France

[d] Departamento de Ciencia de los Materiales e Ingeniería Metalúrgica y Química Inorgánica, Facultad de Ciencias, Universidad de Cádiz, Campus Río San Pedro, 11510 Puerto Real, Spain.

**Supporting Information Description**

Chemical composition and cell parameters of 6H-$BaFeO_{2.96}$, 6H-$BaFeO_{2.90}$ and 6H-$BaFeO_{2.78}$. XPS data of 6H-$BaFeO_{2.96}$, 6H-$BaFeO_{2.90}$ and 6H-$BaFeO_{2.78}$. EELS data of 6H-$BaFeO_{2.96}$ and 6H-$BaFeO_{2.78}$. Neutron diffraction insets and Rietveld refinement and structural parameters of the reduction and oxidation steps. Rietveld refinement data for the revisited $BaFeO_{2.78}$ neutron data under the new orthorhombic symmetry. SAED and HRTEM data during the reduction process of 6H-$BaFeO_{2.96}$ and simulated SAED for the tentative structural model. SEM images of 6H-$BaFeO_{2.96}$, 6H-$BaFeO_{2.90}$ and 6H-$BaFeO_{2.78}$. Physisorption isotherms and BET values for 6H-$BaFeO_{2.90}$ and 6H-$BaFeO_{2.78}$. Catalytic activity of 6H-$BaFeO_{2.96}$.

**Table of contents:**

**1. Chemical analysis and XRD of prepared samples**

**2. X-Ray photoelectron spectroscopy**

**3. EELS spectra**

**4. Neutron diffraction**

**5. SAED and Electron Microscopy**

**6. Morphological and textural properties**

**7. Catalytic activity**

**8. References**

1. **Chemical analysis and XRD of prepared samples**

**Table S1**. Chemical composition and cell parameters of prepared samples.

| Sample | Fe (III) (%) | Fe (IV) (%) | Composition | a (Å) | c (Å) |
|---|---|---|---|---|---|
| **$BaFeO_{2.96}$** | 8 | 92 | $BaFe^{3+}_{0.08}Fe^{4+}_{0.92}O_{2.96}$ | 5.66673(4) | 13.88747(19) |
| **$BaFeO_{2.90}$** | 20 | 80 | $BaFe^{3+}_{0.20}Fe^{4+}_{0.80}O_{2.90}$ | 5.67439(14) | 13.91108(64) |
| **$BaFeO_{2.78}$** | 44 | 56 | $BaFe^{3+}_{0.44}Fe^{4+}_{0.56}O_{2.78}$ | 5.67793(8) | 13.97829(26) |

## 2. X-Ray photoelectron spectroscopy

Figure S1 shows the core level spectra of Ba 3d, O 1s and Fe 2p of the three Ba-Fe samples. The Ba 3d spectra reveal clear splitting of the signals in $BaFeO_{2.90}$ and $BaFeO_{2.96}$ samples, suggesting carbonation of every sample's surface. The formation of carbonates due to the capture of atmospheric $CO_2$ is common in barium and strontium-containing materials due to their high basicity [2, 3]. The O 1s spectra confirm the presence of barium carbonate by a major peak present at 531.4 eV [4, 5] and surface water physisorption is also evidenced by the presence of a small peak (533.3 eV) [6]. Two different Fe signals, correspondent to $2p^{3/2}$ $Fe^{3+}$ (710.4 eV) and $2p^{3/2}$ $Fe^{4+}$ (711.8 eV) can be identified in the broad Fe $2p^{3/2}$ signal at ca. 710.9 eV [7, 8], confirming the presence of mixed oxidation states in the $BaFeO_{3-\delta}$ phases. A higher amount of $Fe^{4+}$ (ca. 60 %) is present in $BaFeO_{2.96}$ and $BaFeO_{2.90}$ while the $BaFeO_{2.80}$ sample exhibits close to 50 % $Fe^{4+}$. The calculated oxidation states of $BaFeO_{3-\delta}$ samples from the Fe 2p spectra are the following: $BaFeO_{2.80}$ - 3.51, $BaFeO_{2.90}$ – 3.54, $BaFeO_{2.96}$ – 3.58. These values may suggest the $BaFeO_{3-\delta}$ samples are slightly reduced in the surface in comparison to the bulk oxidation state determined by other techniques but could also come from the high vacuum from the XPS instrument removing the already proven labile surface oxygen. Oxygen vacancies determination by XPS is a highly controversial topic and while the classical interpretation of the signal at ≈+1 eV $O^{2-}$ O1s signal assigns it to oxygen vacancies [9], new calculations have determined that such assignment is not correct [10, 11]. As these authors also explain, this band may be assigned to surface hydroxyl groups generated by absorption of water in the previous oxygen vacancies sites and that may or may not be quantitively related to oxygen vacancies. We have decided to label our O 1s signal at ≈ 530 eV as O* 1s. It is also noticeable that in our samples' spectra, the intensity of this peak (both with and without deconvolution) can be qualitatively related to the oxygen content detected in the bulk.

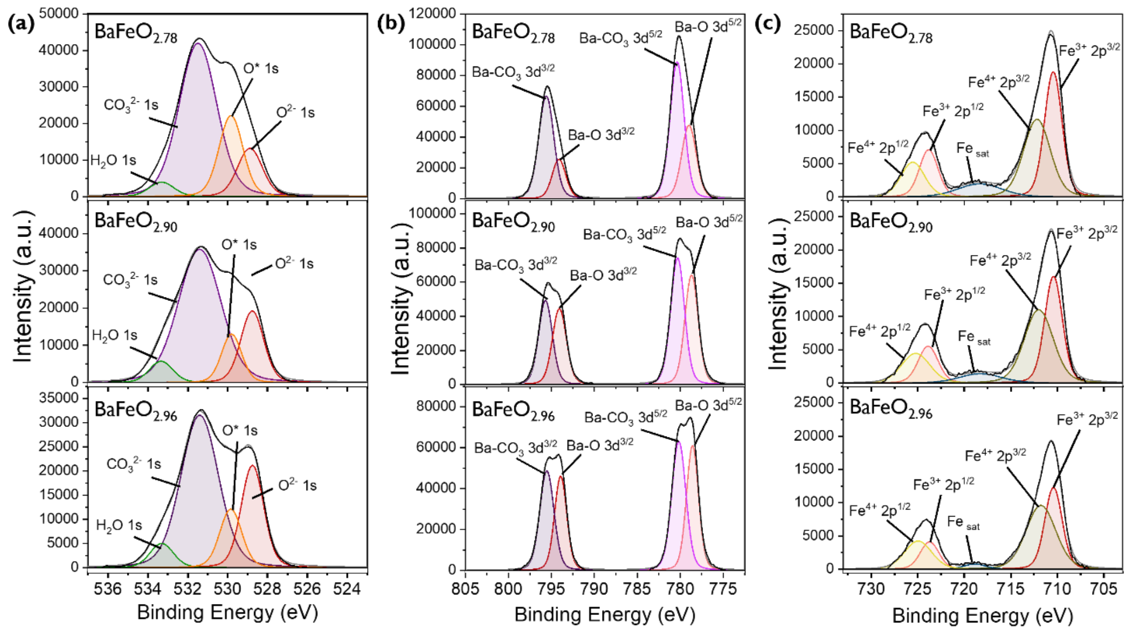


**Figure S1.** Ba 3d (a), O 1s (b), and Fe 2p (c) regions of XPS spectra of $BaFeO_{2.78}$, $BaFeO_{2.96}$ and $BaFeO_{2.90}$ samples

## 3. EELS spectra

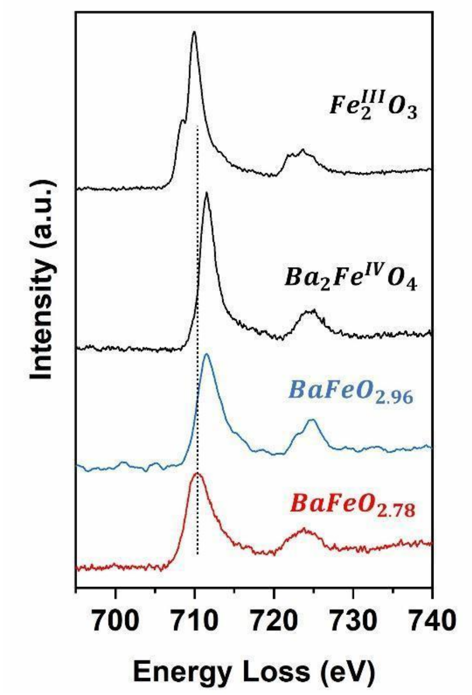


**Figure S2**. EELS Fe-$L_{2,3}$ edges of references with Fe(III), and Fe(IV) oxidation states (black lines); $BaFeO_{2.96}$ (blue) and previously reported $BaFeO_{2.78}$ (red) [1] are shown for comparison.

4. **Neutron diffraction.**

- ***Reduction Process (in vacuum)***

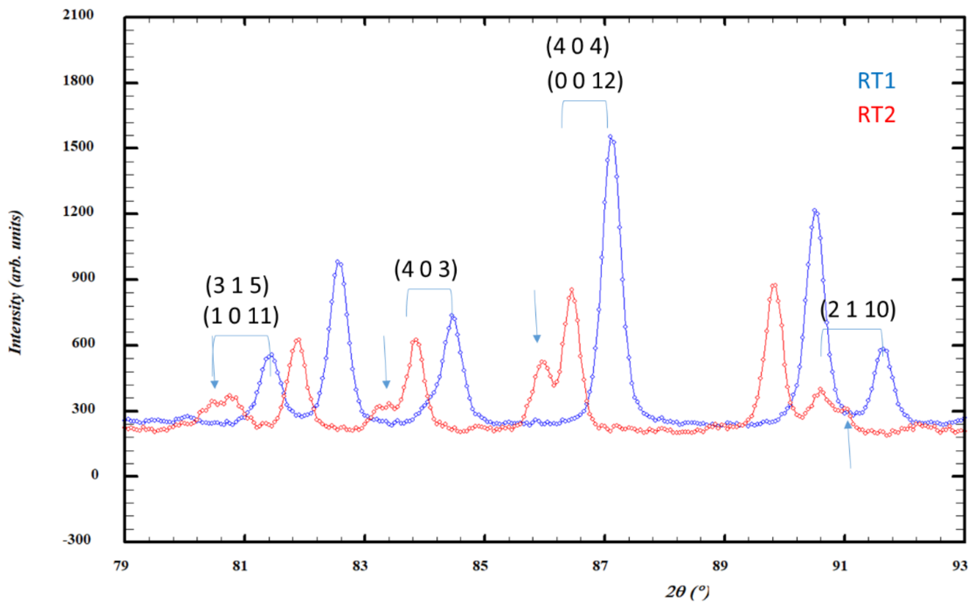


**Figure S3**. ND pattern in the 79 -93° 2θ range of pristine $BaFeO_{2.96}$ (blue) and the sample after cooling down to room temperature under vacuum (RT2). Several maxima split.

**Table S2**. Crystallographic parameters refined from the NDP data for samples O-$BaFeO_{2.669}$ (350°C in vacuum) and O-$BaFeO_{2.651}$ (550°C in vacuum).

| **350 °C O-$BaFeO_{2.669(17)}$** | **x** | **y** | **z** | **Biso (Å$^2$)** | **Occ** |
|---|---|---|---|---|---|
| *Ba1* | 0 | 0.0003(2) | 0.25 | 1.08(3) | 1 |
| *Ba2* | 0.5 | 0.8313(13) | 0.5899(2) | 1.08(3) | 1 |
| *Fe1* | 0 | 0 | 0 | 0.90(2) | 1 |
| *Fe2* | 0.5 | 0.8265(6) | 0.15232(11) | 0.90(2) | 1 |
| *O1* | 0.288(2) | 0.9702(9) | 0.25 | 1.33(3) | 0.78(1) |
| *O2* | 0 | 0.4960(13) | 0.25 | 1.33(3) | 1 |
| *O3* | 0.7422(14) | 0.9139(10) | 0.4152(5) | 1.33(3) | 0.864(8) |
| *O4* | 0 | 0.1698(11) | 0.4194(8) | 1.328(3) | 1 |
| a = 5.7035(2), b= 9.8755(4), c = 14.01552(13) Å. Orthorhombic symmetry *Cmmc* | | | | | |
| $R_B$=2.79, $X^2$=4.67; Rp=2.83; $R_{exp}$=2.01 | | | | | |
| **550 °C O-$BaFeO_{2.651(17)}$** | **x** | **y** | **z** | **Biso (Å$^2$)** | **Occ** |
| *Ba1* | 0 | 0.0004(2) | 0.25 | 1.62(9) | 1 |
| *Ba2* | 0.5 | 0.8325 (18) | 0.5903(2) | 1.72(6) | 1 |
| *Fe1* | 0 | 0 | 0 | 0.79(4) | 1 |
| *Fe2* | 0.5 | 0.8367(8) | 0.15014(10) | 0.79(4) | 1 |
| *O1* | 0.2725(17) | 0.7757(9) | 0.25 | 1.30(19) | 0.66(1) |
| *O2* | 0 | 0.4911(14) | 0.25 | 2.2(2) | 1 |
| *O3* | 0.7477(15) | 0.9075(7) | 0.4183(3) | 2.4(1) | 0.910(7) |
| *O4* | 0 | 0.1676(12) | 0.4124(5) | 0.88(12) | 1 |
| a = 5.73413(19), b= 9.9312(3), c = 14.12700(12) Å. Orthorhombic symmetry *Cmmc* | | | | | |
| $R_B$=1.79, $X^2$=2.83; $R_{exp}$=2.0 | | | | | |

- ***Oxidation process (in air atmosphere)***

**Table S3.** Crystallographic parameters refined from the NDP data for samples O-$BaFeO_{2.69}$ O-$BaFeO_{2.71(2)}$ (200°C and 450°C heating in air, respectively) and H- $BaFeO_{2.912(5)}$ (cooling at 300°C in air).

| Heating in air | | | | | |
|---|---|---|---|---|---|
| **200 °C O-$BaFeO_{2.69(016)}$** | **x** | **y** | **z** | **Biso ($Å^2$)** | **Occ** |
| *Ba1* | 0 | 0.001(3) | 0.25 | 1.81(4) | 1 |
| *Ba2* | 0.5 | 0.829(2) | 0.5924(3) | 1.81(4) | 1 |
| *Fe1* | 0 | 0 | 0 | 1.86(3) | 1 |
| *Fe2* | 0.5 | 0.828(6) | 0.15091(13) | 1.86(3) | 1 |
| *O1* | 0.280(3) | 0.7550(18) | 0.25 | 2.06(3) | 0.693(8) |
| *O2* | 0 | 0.484(2) | 0.25 | 2.06(3) | 1 |
| *O3* | 0.7436(16) | 0.9159(11) | 0.4103(4) | 2.06(3) | 0.921(7) |
| *O4* | 0 | 0.1713(13) | 0.4228(4) | 2.06(3) | 1 |
| a = 5.68796(2), b= 9.86657(19), c = 13.98845(16) Å. Orthorhombic symmetry *Cmmc* | | | | | |
| $R_B$=3.42, $X^2$=2.94; $R_{exp}$=1.72 | | | | | |
| **450 °C O-$BaFeO_{2.71(2)}$** | **x** | **y** | **z** | **Biso ($Å^2$)** | **Occ** |
| *Ba1* | 0 | 0.001(2) | 0.25 | * | 1 |
| *Ba2* | 0.5 | 0.8460(13) | 0.5906(4) | * | 1 |
| *Fe1* | 0 | 0 | 0 | * | 1 |
| *Fe2* | 0.5 | 0.8344(14) | 0.1513(2) | * | 1 |
| *O1* | 0.295(2) | 0.760(2) | 0.25 | * | 0.767(12) |
| *O2* | 0 | 0.4966(18) | 0.25 | * | 1 |
| *O3* | 0.7348(15) | 0.9158(13) | 0.4238(3) | * | 0.901(9) |
| *O4* | 0 | 0.1624(15) | 0.4033(5) | * | 1 |
| | | | | | |
| a = 5.71221(3), b= 9.8928(4), c = 14.0316(2) Å. Orthorhombic symmetry *Cmmc* | | | | | |
| * $B_{overall}$ =2.05(3); $R_B$=2.41, $X^2$=4.12; $R_{exp}$=1.68 | | | | | |

| Cooling in air | | | | | |
|---|---|---|---|---|---|
| **300 °C H-$BaFeO_{2.912(5)}$** | **x** | **y** | **z** | **Biso ($Å^2$)** | **Occ** |
| *Ba1* | 0 | 0 | 0.25 | * | 1 |
| *Ba2* | 0.33333 | 0.66666 | 0.5951(2) | * | 1 |
| *Fe1* | 0 | 0 | 0 | * | 1 |
| *Fe2* | 0.33333 | 0.66666 | 0.15097(13) | * | 1 |
| *O1* | 0.4814(5) | 0.9624(10) | 0.25 | * | |
| *O2* | 0.17230(5) | 0.3459(10) | 0.4163(12) | * | 1 |
| a = 5.69401(4), c = 13.98128(14) Å. Hexagonal symmetry *$P6_3/mmc$* | | | | | |
| * $B_{overall}$ =1.63(3); $R_B$=3.53, $X^2$=2.82; $R_{exp}$=2.02 | | | | | |

**Table S4.** Bond distances and octahedral distortion for orthorhombic O-$BaFeO_{2.69}$ (200 °C in air) and O-$BaFeO_{2.71}$ (450°C in air) and hexagonal H-$BaFeO_{2.912}$ (300°C in air) samples.

| | | **Heating** | | **Cooling** | |
|---|---|---|---|---|---|
| **200 °C** O-$BaFeO_{2.69(016)}$ | | **450 °C** O-$BaFeO_{2.71(2)}$ | | **300 °C** H-$BaFeO_{2.912(5)}$ | |
| Fe1-O octahedra | d(Å) | Fe1-O octahedra | d(Å) | Fe1-O octahedra | d(Å) |
| Fe1 -O3<br>Fe1-O4<br>Average distance: | 2.095(3)x4<br>2.006(4)x2<br>2.065(4) | Fe1 -O3<br>Fe1-O4<br>Average distance | 2.032(4)x4<br>2.103(3)x2<br>2.056(4) | Fe1 –O2 | 2.070(4)x6 |
| Distortion: 4.171 x $10^{-4}$ | | Distortion: 2.620 x $10^{-4}$ | | Distortion: 0.004 x $10^{4}$ | |
| Fe2-O octahedra | d(Å) | Fe2-O octahedra | d(Å) | Fe2-O octahedra | d(Å) |
| Fe2 -O1<br>Fe2-O2<br>Fe2-O3<br>Fe2-O4<br>Average distance | 2.002(3)x2<br>2.070(4)<br>1.884(6)x2<br>1.860(7)<br>1.937(4) | Fe2 -O1<br>Fe2-O2<br>Fe2-O3<br>Fe2-O4<br>Average distance | 1.958(4)x2<br>2.120(3)<br>1.886(6)x2<br>1.866(6)<br>1.945(6) | Fe2 -O1<br>Fe2-O2<br>Average distance | 2.011(4)x3<br>1.839(7)x3<br>1.925(1) |
| Distortion: 21.870 x $10^{-4}$ | | Distortion: 19.444 x $10^{-4}$ | | Distortion: 19.927 x $10^{-4}$ | |

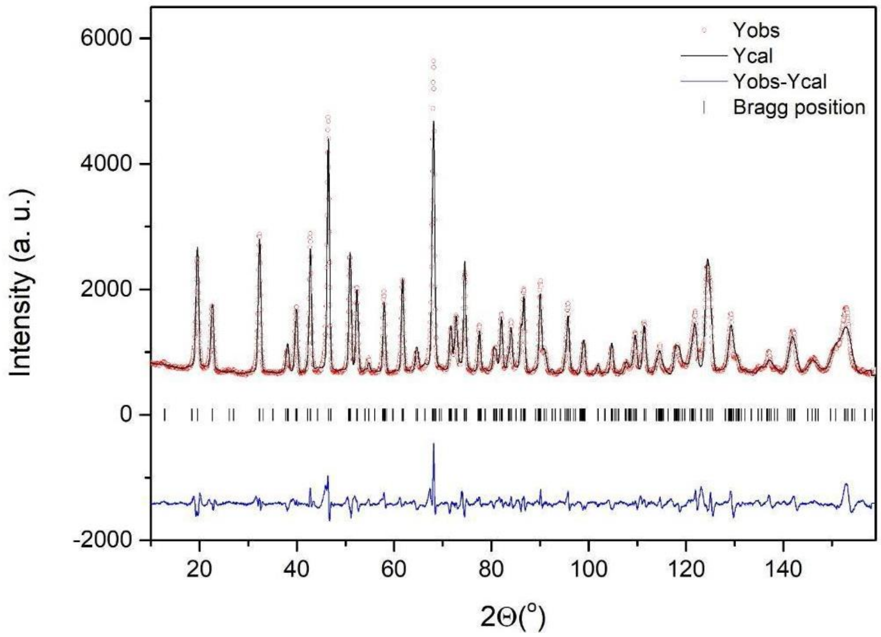


**Figure S4**. Final Rietveld refinement of the NPD data for the revisited 6H-$BaFeO_{2.78}$ [1] in *Cmcm* space group. The observed patterns (red circles), calculated patterns (continuous black line) and difference curves (continuous blue line) are shown.

**Table S5**. Crystallographic parameters refined from the published NDP data for sample 6H-$BaFeO_{2.78(1)}$ (Deposition Number: 2340075)

| **O-$BaFeO_{2.75(3))}$** | **x** | **y** | **z** | **Biso** (Å$^2$) | **Occ** |
|---|---|---|---|---|---|
| *Ba1* | 0 | 0 | 0.25 | 0.46(7) | 1 |
| *Ba2* | 0.5 | 0.838(2) | 0.5888(4) | 0.46(7) | 1 |
| *Fe1* | 0 | 0 | 0 | 0.67(4) | 1 |
| *Fe2* | 0.5 | 0.8329(13) | 0.15355(19) | 0.67(4) | 1 |
| *O1* | 0.278(2) | 0.8329(19) | 0.25 | 0.68(5) | 0.91(9) |
| *O2* | 0 | 0.4732(17) | 0.25 | 0.68(5) | 1 |
| *O3* | 0.734(3) | 0.9194(16) | 0.415(3) | 0.68(5) | 0.86(1) |
| *O4* | 0 | 0.161(2) | 0.4228(5) | 0.68(5) | 1 |
| a = 5.67195(4), b= 9.8291(7) c = 13.9824(3) Å. Orthorhombic symmetry *Cmmc* | | | | | |
| $R_B$=6, $X^2$=4.46; $R_{exp}$=3.55 | | | | | |

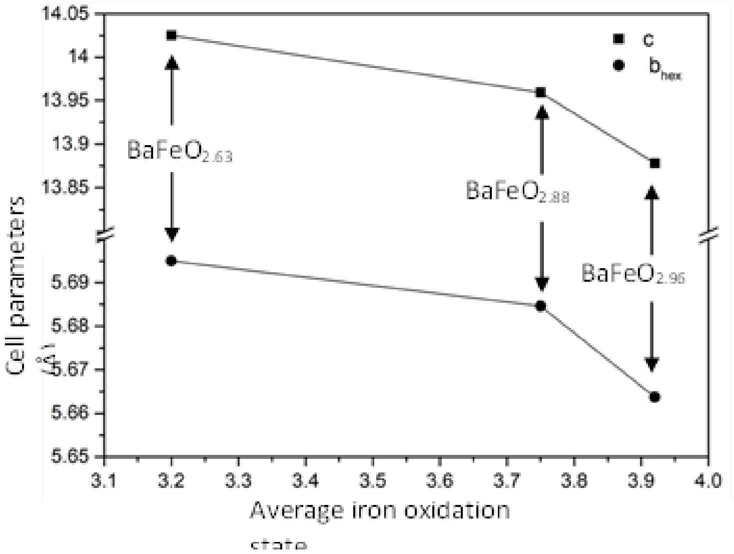


**Figure S5.** Changes in lattice parameters (referred to the hexagonal cell) as a function of oxygen composition in $BaFeO_{3-\delta}$

## 5. SAED and Electron Microscopy

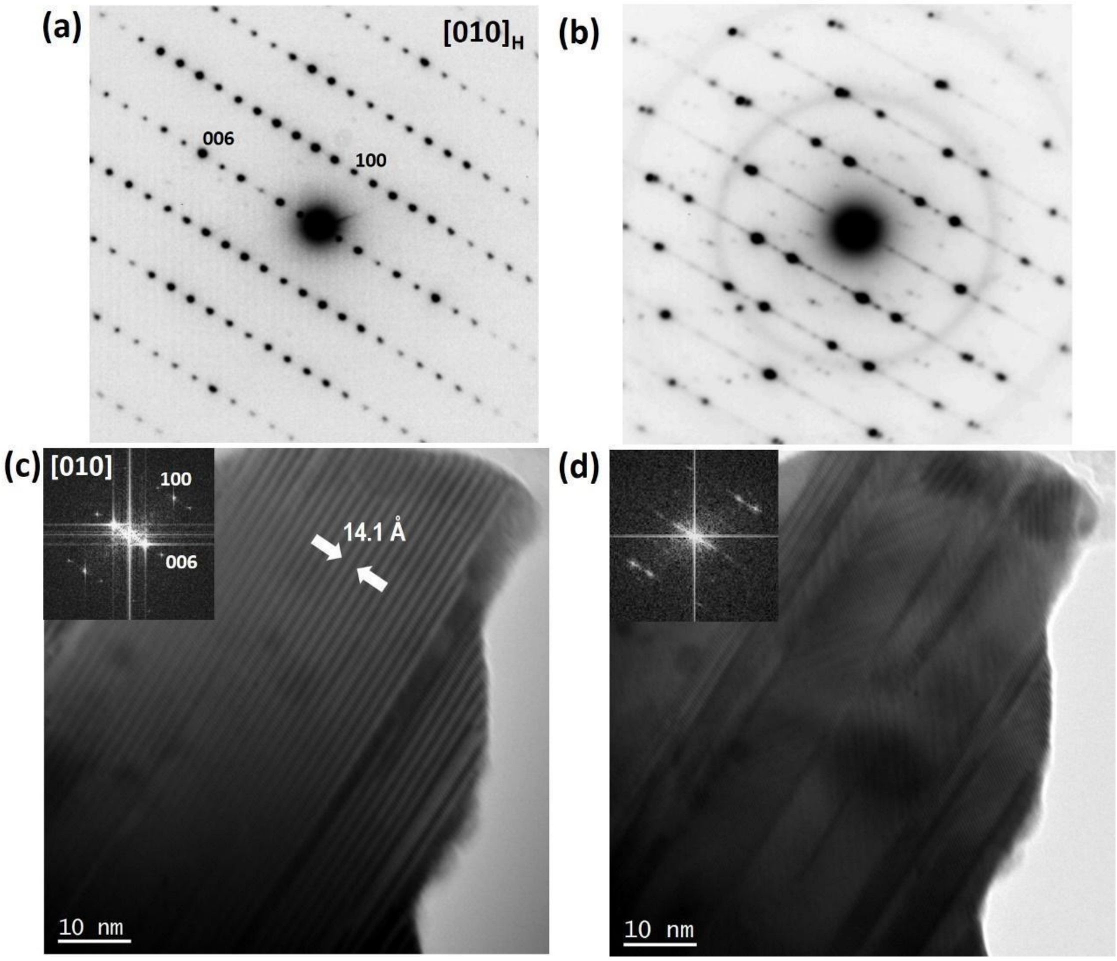


**Figure S6**. Electron diffraction patterns along [010] of the H-$BaFeO_{2.96}$ sample recorded at **(a)** 40 °C and **(b)** 550 °C. Corresponding TEM images are shown in Figures **(c)** and **(d)**. At 550 °C the 6H packing sequence along the c axis ($d_{001}$ = 14.1 Å) is lost irreversibly. Notice the drift in both images because of the vibration due to the water flow of the heating stage.

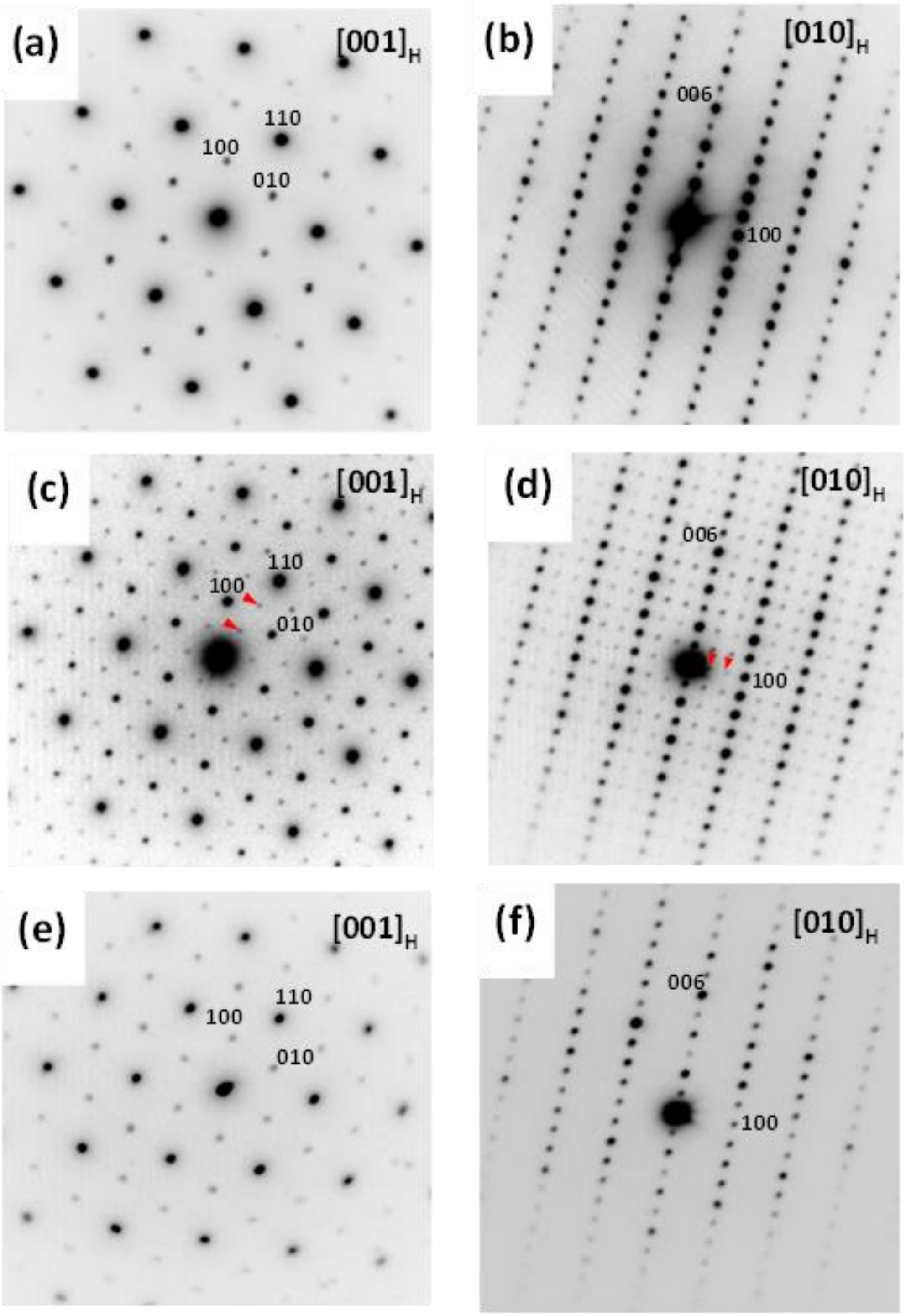


**Figure S7**. Electron diffraction patterns along [001] and [010] of the H-$BaFeO_{2,96}$ sample recorded as a function of the temperature: **(a), (b) at** 25 ºC; **(c), (d)** at 300 ºC; and **(e), (f)** at 25 ºC after cooling.

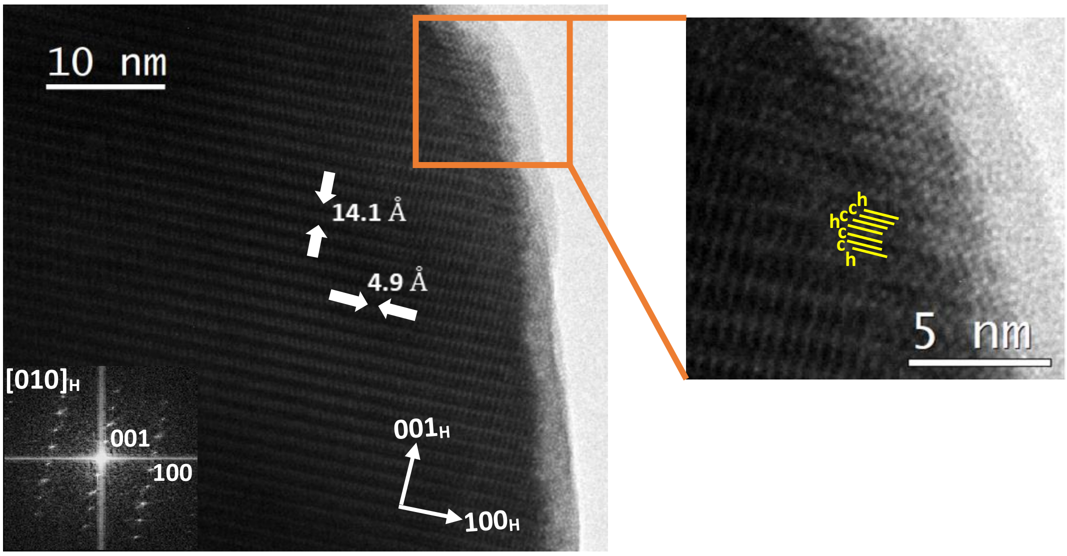


**Figure S8**. TEM image along [010] of the H-$BaFeO_{2.96}$ sample recorded at 25 °C after cooling. Distances of 14.1 and 4.9 Å can be measured along $001_H$ and $100_H$ respectively. Corresponding FFT is shown as inset. The enlarged image of the thinnest region of the crystals (marked in orange) allows to identify the –cchcch- sequence of the 6H polytype.

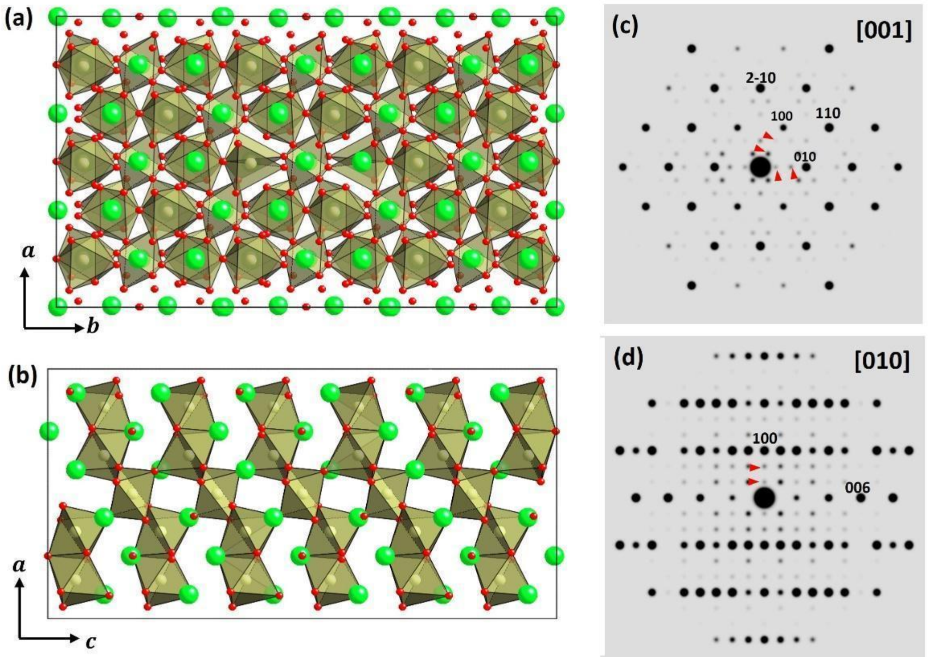


**Figure S9** Simulated **(c)** [001] and **(d)** [010] SAED diagrams are obtained considering a tentative structural model **(a, b)** (C2 space group; a=16.81, b=28.87 and c=14.18 Å) using as input the highly distorted 6H framework previously described for $SrIrO_3$ [12] and introducing anionic vacancies to get the three-fold order in *a* and *b* axes experimentally observed (marked by red arrows). In this model, the octahedral arrangement is distorted in a crooked way comparing with the pristine 6H structure.

## 6. Morphological and textural properties

Figure S10 corresponds to SEM images of the $BaFeO_{2.78}$, $BaFeO_{2.90}$ and $BaFeO_{2.96}$ samples. The first two micrographs show particles of similar size, ranging from 100 to 200 nm. However, the most oxidized $BaFeO_{2.96}$ sample clearly shows a higher degree of sintering thus, the particle size is larger and not in the same range as the other samples.

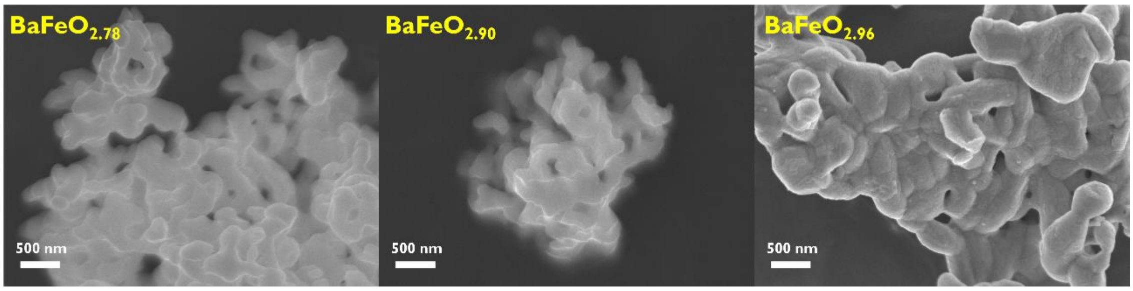


**Figura S10.** SEM micrographs corresponding to $BaFeO_{2.78}$, $BaFeO_{2.90}$ and $BaFeO_{2.96}$ samples.

Table S6 summarizes the main parameters derived from the measured isotherms. Low values of BET surface and pore volume are related to the synthesis method employed to prepare the solids. The presence of micropores can be excluded in all the samples studied. In all cases, the isotherms can be described as type II according to the IUPAC indications. Considering the results in Fig. S11, these pores can be described as mesopores.

**Table S6**. Textural properties obtained from $N_2$ physisorption at −196 ºC measurements.

| Sample | BET specific surface area ($m^2$ $g^{-1}$) [a] | Average pore diameter (nm) [b] | Total pore volume ($cm^3$ $g^{-1}$) [c] |
|---|---|---|---|
| $BaFeO_{2.78}$ | 3.3 | 19.9 | 0.007 |
| $BaFeO_{2.90}$ | 3.8 | 17.8 | 0.015 |

[a] Calculated by BET method.
[b] Determined by BJH method using the desorption branch.
[c] Determined from the amount adsorbed at P/P0 = 0.99.

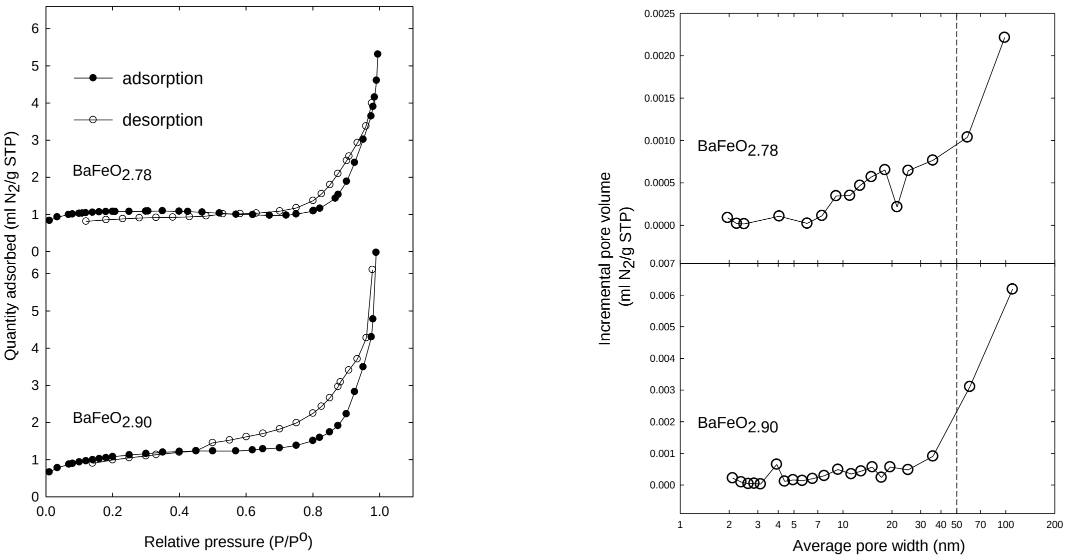


**Figure S11.** Nitrogen physisorption isotherm registered at −196 ºC, adsorption (•) and desorption (o) branches are shown (left). Incremental pore size distribution curves by means of BJH analysis of the desorption branch of the $N_2$ isotherm at-196 ºC (right).

## 7. Catalytic activity

Figure **S12** shows the CO conversions for $BaFeO_{2.78}$, $BaFeO_{2.90}$, and $BaFeO_{2.96}$ perovskites. $BaFeO_{2.96}$ perovskite was obtained by high-temperature oxidation at the expense of a low specific surface area. Therefore, despite having a higher $Fe^{4+}$ content, the CO conversion was similar to that of the $BaFeO_{2.78}$ perovskite.

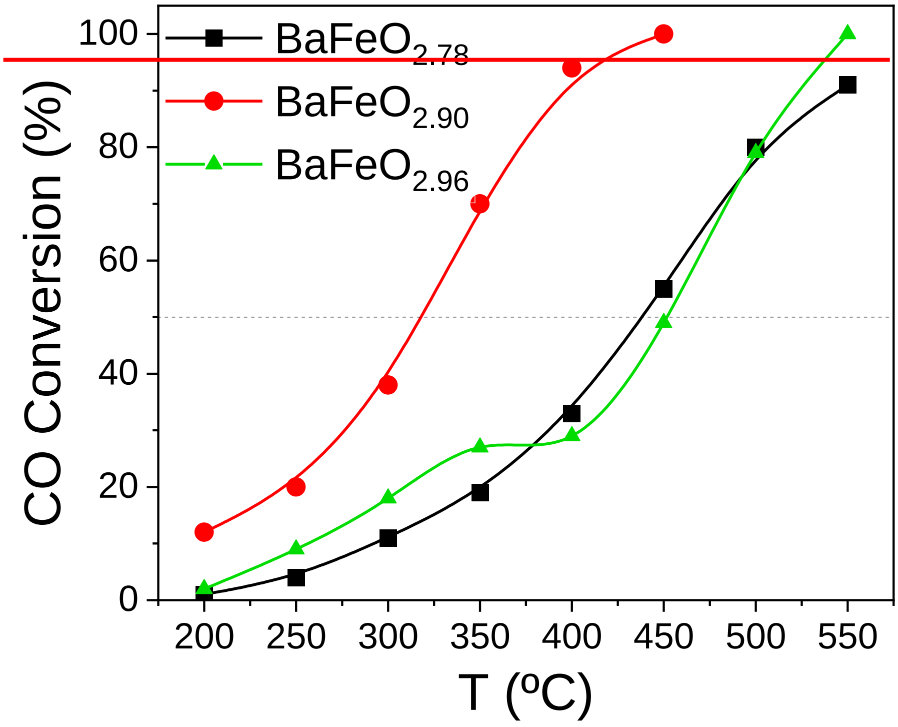


**Figure S12.** Light−off curves of CO conversion for CO oxidation reaction over $BaFeO_{2.78}$, $BaFeO_{2.90}$ and $BaFeO_{2.96}$ perovskites.

## 8. References:

[1]El-Hadri, A., Gómez-Recio, I., del Rio, E., Hernández-Garrido, J.C., Cortés-Gil, R., Hernando, M., Varela, A., Gutiérrez-Alonso, A., Parras, M., Delgado J.J., Pérez-Omil, J.A., Blanco, G., Calvino, J.J. and González-Calbet, J.M. *Critical influence of redox pretreatments on the CO oxidation activity of* $BaFeO_{3-\delta}$ *perovskites: an in-depth atomic-scale analysis by aberration-corrected and in situ diffraction techniques.* ACS Catalysis (2017), 7,12, 8653–8663.

[2]Chen, C., Chen, D., Gao, Y., Shao, Z. and Ciucci, F. *Computational and experimental analysis of* $Ba_{0.95}La_{0.05}FeO_{3-\delta}$ *as a cathode material for solid oxide fuel cells*. Journal of Materials Chemistry A (2014), 2(34), 14154-14163.

[3]Falcón, H., Barbero, J. A., Alonso, J. A., Martínez-Lope, M. J. and Fierro, J. L. G. $SrFeO_{3-\delta}$ *perovskite oxides: chemical features and performance for methane combustion*. Chemistry of Materials (2002),14(5), 2325-2333.

[4]Schmitz, P. J. *Characterization of the Surface of* $BaCO_3$ *Powder by XP*S. Surface Science Spectra (2001), 8(3), 190-194.

[5]Dissanayake, D. P., Kharas, K. C. C., Lunsford, J. H. and Rosynek, M. P. *Catalytic partial oxidation of methane over Ba-Pb, Ba-Bi, and Ba-Sn perovskites.* Journal of Catalysis (1993), 139(2), 652-663.

[6]Nefedov, V. I., Gati, D., Dzhurinskii, B. F., Sergushin, N. P. and Salyn, Y. V. *X-ray electron study of oxides of elements.* Zhurnal Neorganicheskoi Khimii (1975), 20(9), 2307-2314.

[7]Wang, J., Saccoccio, M., Chen, D., Gao, Y., Chen, C. and Ciucci, F. *The effect of A-site and B-site substitution on* $BaFeO_{3-\delta}$*: an investigation as a cathode material for intermediate-temperature solid oxide fuel cells*. Journal of power sources (2015), 297, 511-518.

[8] Ghaffari, M., Shannon, M., Hui, H., Tan, O. K. and Irannejad, A. *Preparation, surface state and band structure studies of* $SrTi_{(1-x)}Fe_{(x)}O_{(3-\delta)}$*(x= 0-1) perovskite-type nano structure by X-ray and ultraviolet photoelectron spectroscopy*. Surface science (2012), 606(5-6), 670-677.

[9] Abdel-Khalek, E. K., Motawea, M. A., Aboelnasr, M. A. and El-Bahnasawy, H. H. *Study the oxygen vacancies and Fe oxidation states in* $CaFeO_{3-\delta}$ *perovskite nanomaterial.* Physica B: Condensed Matter (2022), 624, 413415.

[10] Idriss, H. *On the wrong assignment of the XPS O1s signal at 531–532 eV attributed to oxygen vacancies in photo-and electro-catalysts for water splitting and other materials applications.* Surface Science (2021), 712, 121894.

[11] Frankcombe, T. J. and Liu, Y. *Interpretation of Oxygen 1s X-ray Photoelectron Spectroscopy of ZnO.* Chemistry of Materials (2023), 35, 14, 5468-5474.

[12] Krombo C.H., Nielsen M.B., Kevy, S.M., Parisiades, P. and Bremholm M. *High pressure structure studies of 6H-*$SrIrO_3$ *and the octahedral tilting in 3C-*$SrIrO_3$ *towards a post-perovskite.* Journal of Solid State Chemistry (2016), 238, 74–82
